# Irisin attenuates vascular remodeling in hypertensive mice induced by Ang II by suppressing Ca^2+^-dependent endoplasmic reticulum stress in VSMCs

**DOI:** 10.7150/ijbs.84153

**Published:** 2024-01-01

**Authors:** Ru-li Li, Cai-li Zhuo, Xin Yan, He Li, Lan Lin, Ling-yu Li, Qiying Jiang, Die Zhang, Xue-mei Wang, Lin-ling Liu, Wen-jing Huang, Ying-ling Wang, Xin-yue Li, Yan Mao, Yixin Chen, Xiao Liu, Quan-chen Xu, Yu-yan Cai, Xi-jing Yang, Hong-ying Chen, Si-si Wu, Wei Jiang

**Affiliations:** 1 Molecular Medicine Research Center, State Key Laboratory of Biotherapy,West China Hospital, Sichuan University, Chengdu, Sichuan 610041, PR China.; 2 Sichuan Key Laboratory of TCM Regulating Metabolic Diseases, Affiliated Hospital of Chengdu University of Traditional Chinese Medicine, Chengdu University of Traditional Chinese Medicine, Chengdu, Sichuan, 610072, PR China.; 3 West China College of Preclinical Medicine and Forensic Medicine, Sichuan University, Chengdu, Sichuan 610041, PR China.; 4 Department of Biomedical Engineering, Southern University of Science and Technology, Guangdong 518055, PR. China.; 5 Department of Cardiology, West China Hospital, Sichuan University, Chengdu, Sichuan 610041, PR China.; 6 Core Facilities, West China Hospital, Sichuan University, Chengdu, Sichuan 610041, PR China.

**Keywords:** Hypertension, Vascular remodeling, Irisin, Calcium homeostasis, ERS

## Abstract

Vascular remodeling plays a vital role in hypertensive diseases and is an important target for hypertension treatment. Irisin, a newly discovered myokine and adipokine, has been found to have beneficial effects on various cardiovascular diseases. However, the pharmacological effect of irisin in antagonizing hypertension-induced vascular remodeling is not well understood. In the present study, we investigated the protection and mechanisms of irisin against hypertension and vascular remodeling induced by angiotensin II (Ang II). Adult male mice of wild-type, *FNDC5* (irisin-precursor) knockout, and *FNDC5* overexpression were used to develop hypertension by challenging them with Ang II subcutaneously in the back using a microosmotic pump for 4 weeks. Similar to the attenuation of irisin on Ang II-induced VSMCs remodeling, endogenous *FNDC5* ablation exacerbated, and exogenous *FNDC5* overexpression alleviated Ang II-induced hypertension and vascular remodeling. Aortic RNA sequencing showed that irisin deficiency exacerbated intracellular calcium imbalance and increased vasoconstriction, which was parallel to the deterioration in both ER calcium dysmetabolism and ER stress. *FNDC5* overexpression/exogenous irisin supplementation protected VSMCs from Ang II-induced remodeling by improving endoplasmic reticulum (ER) homeostasis. This improvement includes inhibiting Ca^2+^ release from the ER and promoting the re-absorption of Ca^2+^ into the ER, thus relieving Ca^2+^-dependent ER stress. Furthermore, irisin was confirmed to bind to its receptors, αV/β5 integrins, to further activate the AMPK pathway and inhibit the p38 pathway, leading to vasoprotection in Ang II-insulted VSMCs. These results indicate that irisin protects against hypertension and vascular remodeling in Ang II-challenged mice by restoring calcium homeostasis and attenuating ER stress in VSMCs via activating AMPK and suppressing p38 signaling.

## 1. Introduction

Hypertension, the most common modifiable risk factor for early cardiovascular diseases and cardiovascular mortality, is characterized by elevated arterial pressure, arterial resistance and stiffness, as well as targeted organ damage^1^, ^2^, ^3^. A critical pathological feature of hypertension is the occurrence of vascular remodeling, which results from functional and structural alterations that emerge in both large and small arteries. This is accompanied by vascular wall thickening, erosion of the lumen, an increase in the media/lumen ratio, a decrease in the number of tiny arteries, and subsequently, the emergence of vascular dysfunction^3^. Vascular remodeling is a key common pathological basis that further leads to functional or structural changes in heart, blood vessel, brain, and kidney, resulting in the hypertensive complications^2^, ^3^. Vascular stiffness is a significant pathological change in hypertension that results from extracellular matrix (ECM) secretion and vascular fibrosis 4, 5. Vascular smooth muscle cells (VSMCs) has an important role in regulating the vascular remodeling process 3. In addition to their contractile properties, VSMCs contribute substantially to vascular remodeling through abnormal proliferation, migration, and phenotypic transformation from the contractile to the synthetic phenotype 3, 5. The VSMC phenotype's plasticity makes these cells particularly adept at stimulating myofibroblasts to produce ECM 5. VSMCs live in a liquid internal environment composed of various cytokines, protein hormones, extracellular enzymes, ions and gases such as oxygen and carbon dioxide^3^. Among them, vasoactive factors, including atrial natriuretic peptide, adiponectin and adrenomedullin, maintain the vascular functional homeostasis and regulate vascular remodeling well by controlling the growth, migration and fate of VSMCs, as well as regulating the matrix components and inflammation in the vascular wall^6^.

Irisin, a novel exercise-induced hormone-like myokine, deriving from fibronectin type III domain-containing protein 5 (*FNDC5*) on the cell membrane through protease-induced hydrolysis and modification 7, 8. It has been identified as a new vasoactive factor 7. Circulating irisin is primarily derived from skeletal muscle and myocardium, and it is widely expressed in various tissues and organs 9. Irisin is closely associated with the occurrence and development of various metabolic disorders and plays a protective role in regulating cellular metabolism 7-11. Growing evidence suggested that irisin provided direct endogenous cardiovascular protection^12^-^14^. It attenuated atherosclerosis by improving endothelial function, reducing the formation of foam cells, and inhibiting the inflammation^13^. It also protected against myocardial ischemia/reperfusion and hypoxia/reoxygenation injury, via promoting the endogenous antioxidant capacity, mitochondrial function and angiogenesis, but suppressing oxidative stress and apoptosis, and activating mitophagy^15^. Our previous studies have further confirmed that irisin protects the heart from hypertrophy by inducing autophagy and autophagic flux through the activation of the AMPK-ULK1 signaling pathway 16. Irisin has been determined to have a significant impact on hypertension 12. In hypertensive patients, irisin levels are often negatively correlated with systolic and diastolic blood pressure 17. In the central nervous system, irisin reduces blood pressure by suppressing the activity of paraventricular nuclei through activating the nuclear factor Nrf2 signaling pathway 18. In peripheral tissues, irisin relaxes hypertensive mesenteric arteries in an endothelium-dependent manner. It does so by activating the NO-cGMP-dependent pathway to promote transient receptor potential vanilloid 4 (TRPV4) channel-induced extracellular calcium influx or the AMPK-Akt-NO signaling axis to attenuate endothelial dysfunction 12, 19, 20. Additionally, irisin relaxes arteries in an endothelium-independent manner by inhibiting Ca^2+^ influx and activating ATP-sensitive potassium channels 18. However, the pathogenesis of hypertension is so complex, and the regulation of irisin on VSMCs and the tunica media fibrosis in hypertensive arteries is unclear.

The calcium levels within VSMCs regulate their phenotype and function by determining their contractile state and influencing the activity of various calcium-dependent proteins and transcription factors 21. Dysregulation of calcium signaling and VSMC function can lead to increased vascular resistance and the development of hypertension by interfering with vascular reactivity and tone 22. Calcium homeostasis in VSMCs depends on a series of complex mechanisms that regulate cellular calcium influx and efflux, as well as intracellular calcium storage. 23. The critical physiological functions of the endoplasmic reticulum (ER) are to store and release intracellular Ca^2+ 24^. The disturbance of ER homeostasis induces unfolded protein response (UPR) in an attempt to restore cell equilibrium during ER stress^25^. Sustained ER stress, and inadequate compensation for it, leads to VSMC injury, a process associated with VSMC remodeling in hypertension^26^. Evidence suggests that Ca^2+^ signaling is crucial for the ER stress response, as it is involved in many cellular activities 27. These studies indicate that calcium mediate the regulation of ER on VSMC functions^25^-^27^. Thus, we hypothesized that irisin, a new vasoactive molecule, may attenuate vascular remodeling in hypertension by sophisticatedly regulating ER function in VSMCs. We discovered that irisin prevented VSMCs from hypertension and vascular remodeling caused by Ang II by enhancing intracellular calcium homeostasis and encroaching calcium-dependent ER stress through activating AMPK and suppressing p38 signaling pathways via αV/β5 receptors on the membrane. This study is the first to clarify the protection and mechanisms of irisin in vascular remodeling by regulating ER function, potentially providing new biomarkers and pharmacological targets for chronic vascular remodeling in hypertension.

## 2. Materials and Methods

### 2.1. Chemicals and reagents

Irisin was purchased from Cayman Chemical (Ann Arbor, MI, USA). Angiotensin II (Ang II), BAPTA-AM and 4-PBA were purchased from Selleck Chemicals (Houston, TX, USA). Alezet Osmotic pump (2004) was purchased from Alzet (Cupertino, CA, USA). Transwell for migration was purchased from Corning (NY, USA). CCK8 was purchased from Biomake (Houston, TX, USA). The irisin ELISA kits were purchased from Phoenix Pharmaceuticals Inc. (Burlingame, CA, USA). Compound C and HY-W007355 were purchased from MedChemExpress (NJ, USA). Edu cell proliferation kit, Fluo-3 AM Calcium probe and Crystal violet dye were obtained from Beyotime Biotechnology (Shanghai, China). anti-RYR2 was purchased from Abcam (Waltham, MA, USA); anti-IP3R, anti-SERCA, anti-p-SERCA, anti-PLN, anti-p-PLN were obtained from Abclonal (Wuhan, China); anti-p-CaMK II, anti-CaMKII were purchased from Santa Cruz (Dallas, TX, USA); anti-Ki67, anti-Bip, anti-ATF6, anti-IRE1α, anti-p-eif2α, anti-eif2α, anti-p-AMPK, anti-AMPK, anti-p-ERK, anti-ERK, anti-p-p38, anti-p38, anti-p-JNK, anti-JNK and anti-β-tubulin were obtained from Cell Signaling Technology (Danvers, MA, USA); anti-αV/β5 antibody was obtained from Millipore (Boston, MA, USA). All the secondary antibodies used in this study were provided by Zhongshan Golden Bridge Biotechnology (Beijing, China). All chemicals and reagents used were of analytically pure.

### 2.2 Animals

We generated mouse-*FNDC5* (irisin-precursor)-knockout mice (irisin-KO) on a C57BL/6 background with the help of Beijing Biocytogen Co. Additionally, we obtained *FNDC5* transgenic mice (irisin-OV) on a C57BL/6 background from Shanghai Biomodel organism. Wild-type (WT) littermates were used as controls. The genotyping of irisin-KO and WT mice was performed using PCR assays with specific primers (forward: 5′‐CAAGGGACCCTGTTCTGAATGTCCC-3′ and reverse: 5′‐TCCCTCACCTAACTCACCCTCCTTG‐3′), as shown in Supplementary Figure 1, following the method described in our previously published paper 16, 28. Similarly, the genotyping of irisin-OV and WT mice was identified using specific primer sequences (forward: 5′‐TCACCGTCAGGCACCTCAA‐3′ and reverse: 5′‐GTCATACTGGCGGCAGAAGA‐3′). To conduct the experiments, 8-week-old male irisin-KO mice, irisin-OV mice, and matched WT mice were assigned into the sham and Ang II-treated group randomly. Subcutaneously implanted osmotic pumps were used to infuse mice with saline or Ang II (490 ng/min/kg) 29 for 4 weeks, and then the carotid blood pressure was measured, and the thoracic aorta and mesenteric arteries were removed for RNA and protein expression analysis, as well as histological examination to assess hypertension and remodeling. All animal experiments were approved by the Sichuan University Committee on Animal Care in accordance with the National Institutes of Health Guidelines on the Use of Laboratory Animals (Permit Number: 20220615001, date: 20220615).

### 2.3 Blood pressure detection

The caudal arterial pressure of mice was noninvasively measured using a tail-cuff system (BP-2010A; Softron, Tokyo, Japan) at the same time every week, as described previously 30. Briefly, the mice were fixed in a specialized mouse barrel for 10-15 minutes to acclimate to the experimental environment. Subsequently, the caudal arterial pressure was detected 3 times after achieving balance, and the average value was calculated to determine systolic blood pressure (SBP), mean blood pressure (MBP), diastolic blood pressure (DBP), and heart rate (HR). At the endpoint of the experiments, internal carotid artery blood pressure was obtained by intubating the carotid artery and recording the measurements using an eight-channel physiological recorder (iWorx 308, iWorx/CB Sciences, Dover, NH, USA) 30. In summary, the mice were placed in a supine position and underwent carotid artery catheterization after being anesthetized with isoflurane. The carotid artery blood pressure was recorded after a half-hour balance period.

### 2.4 Histology and morphometric analysis

The isolated thoracic aortas and the mesenteric arteries were paraffin-embedded after fixed in 4% neutral formaldehyde and cut into sections of 4 μm thickness. Sections of vascular tissues were stained with either hematoxylin and eosin (H&E), Masson, or Elastin to examine vascular structure, deposition of collagen and elastin, respectively. Images were obtained using a Zeiss AxioCam Microscope (Carl Zeiss, Germany). HE-stained arterial tissues were used to measure the luminal radius and media thickness. The media/lumen ratios were determined according to the previous description 30. Moreover, analysis of the density of collagen and elastin in the vascular wall was obtained using Image-Pro Plus 5.1 (Media Cybernetics, Inc., USA) according to the methods previously described 30. The density of elastin and collagen was measured for the calculation of the ratios of elastin/collagen.

After deparaffinizing thoracic aortic sections (about 4-5 mice per group), they were permeated with 0.02% TritonX-100. Subsequently, the sections were incubated overnight in darkness at 4℃ with a mouse anti-Ki-67 primary antibody (1:100) and an anti-SMC-α-actin antibody (1:150) from Cell Signaling Technology. Ki-67 was visualized by incubating the sections with an Alexa Fluor 594-conjugated secondary antibody (1:500), while SMC α-actin was indicated by incubating with a FITC-conjugated secondary antibody (1:500) for an additional hour under room temperature. Following this, the sections were stained with DAPI for 5 seconds under room temperature and examined using a Zeiss fluorescence microscope (Carl Zeiss, Germany). An investigator who was blinded to the treatment captured Ki-67-positive VSMCs in 5 random fields each section. The Ki-67 labeling indexes were then presented as the average percentage of Ki-67-positively stained VSMCs in the total VSMC count of the aortic media within those 5 fields.

### 2.5 Irisin content assays

An irisin ELISA kit (Phoenix Pharmaceuticals Inc, Burlingame, CA) was used to detect the irisin levels in mouse plasma, following the manufacturer's instructions. Plasma was obtained by centrifugation after collecting blood from the inferior vena cava of anesthetized mice. Subsequently, the plasma was diluted and tested to calculate the irisin levels based on the standard curves created.

### 2.6 RNA-seq

Aorta RNA sequencing was performed on 4-week Ang II-treated WT and irisin-KO mice by Novogene Biotech. In brief, RNA was extracted from aorta tissue to assess its integrity and quantity. The transcriptome sequencing library was constructed and sequenced using the Illumina NovaSeq 6000 system after passing quality inspection. The two comparison combinations were analyzed for differential expression using DESeq. 2 software (version 1. 20. 0). The differential expression of gene expression data was determined using a negative binomial distribution model. A threshold of padj ≤ 0. 05 was set for differential expression. The ClusterProfiler R software package (version 3) was utilized to perform gene ontology (GO) enrichment analyses and KEGG enrichment analyses for the genes with different expression.

### 2.7 Primary VSMCs isolation and culture

A classical enzymatic digestion method was employed to isolate and culture primary mouse VSMCs 30. Briefly, mouse aortas were excised, and any visible fat was removed. The vessels were then placed in enzymatic solution I (2 mg/mL collagenase II) at 37 ℃ for 20 minutes. After the initial digestion, the adventitial tissue was easily removed, and the media tissue was cut into small pieces and placed in enzymatic solution II (0. 5 mg/mL elastase VI and 1 mg/mL collagenase II) for further digestion. After approximately 60 minutes, the tissue digestion was terminated by adding DMEM containing 10% FBS. The digestion solution was centrifugated at 1000 g for 5 minutes to collect cells. The cell precipitates were suspended in warm 20% FBS culture medium and added to petri dishes for further cultivation. The type and purity of cells were determined by immunofluorescence staining using the SMC-specific α-actin antibody.

### 2.8 VSMCs proliferation

The Cell Counting Kit-8 (CCK8) assay 31 and EDU assay 32 were conducted to examine VSMC proliferation, respectively. Mouse VSMCs were counted and seeded in 96-well plates with 5000 cells each well. They were then pretreated with irisin (5, 10, 20 nM) for 30 minutes, and then treated with Ang II (1 μM) for an additional 24 hours. After the specified treatment time, the CCK-8 substituted medium was added in and incubated for 1 hour. The cell viability was detected at 450 nm using a BioTek microplate reader (Winooski, VT).

In the EDU assay, VSMCs were seeded in 24-well plates with 20,000 cells per well. After the drug treatment, the cells were supplemented with 100 μL of 5-ethynyl-2-deoxyuridine (EDU) for another 2 hours. Subsequently, the cells were washed and fixed with paraformaldehyde (4%) for 15 minutes. To develop the EDU color, the cells were incubated with 0. 3% TritonX-100 for 15 minutes, followed by the click reaction solution (50 μL) addition. Additionally, the cell nuclei were stained by Hoechst 33342 (diluted 1:1,000), and the EDU-positive cells were visualized using a fluorescence microscopy.

### 2.9 Transwell of migration

Resuspended VSMCs (about 10^5^ cells) were added to the upper chambers of 8 μm pore size transwell inserts (Corning, USA) with 200 μL fresh medium without FBS. Ang II (1 μM) was co-incubated with or without 20 nM irisin in the cells. The lower chamber was added with cell medium (600 μL) supplemented with 20% FBS. After incubating the cells for 6 hours in a cell incubator, we used a cotton swab to gently scrape off the cells in the upper wells. The cells migrated to the lower membrane surface were fixed in paraformaldehyde (4%) for 15 minutes and then stained with crystal violet. Five randomly selected fields (200× objective) were assigned to each well, and the migrated cell number was manually counted 31. All data were normalized to the migration levels of the control VSMCs.

### 2.10 Western Blotting

We performed western blotting assays following the protocol previously described 33. Treated VSMCs were lysed with RIPA lysis buffer for 30 minutes on ice, and then the supernatants were collected after centrifugation. The BCA protein assays were used to detect the protein concentrations. Samples containing 35 μg of protein were loaded into 10% and 6% SDS PAGE gels per lane and subsequently transferred to PVDF membranes, and then incubated with specific primary antibodies overnight at 4°C after treated with Rapid Block Buffer for 20 min. In the next day, they were immersed in horseradish peroxidase-conjugated secondary antibodies for 1 hour under room temperature. The protein expression levels were detected using an enhanced chemiluminescence (ECL) kit and quantified by scanning densitometry.

### 2.11 q-PCR

We followed the manufacturer's protocol to extract total mRNA from VSMCs and mouse aortic tissues using Trizol reagent (Life Technologies) 28. We used a primeScript RT-PCR Kit for reverse transcription and specific primers to perform semi-quantitative real-time PCR. The DNA targets were further amplified with the primers listed in Table 1. We employed the 2^-ΔΔCT^ method to analyze all data normalized to GAPDH mRNA levels as a reference gene. The results were displayed as control percentages 28.

### 2.12 Statistical analysis

All data were represented as mean ± S.E.M. All the experiments were repeated independently at least three times. GraphPad Prism 7 was performed for statistical analyses. The two experimental groups were compared using a two-tailed unpaired Student's *t*-test. Three or more groups were compared using one-way ANOVA. A p<0.05 was considered as statistically significant.

## 3. Results

### 3.1 The expression of the FNDC5 gene and protein in VSMCs, as well as the irisin contents in VSMC culture supernatants

**Figure 1:**
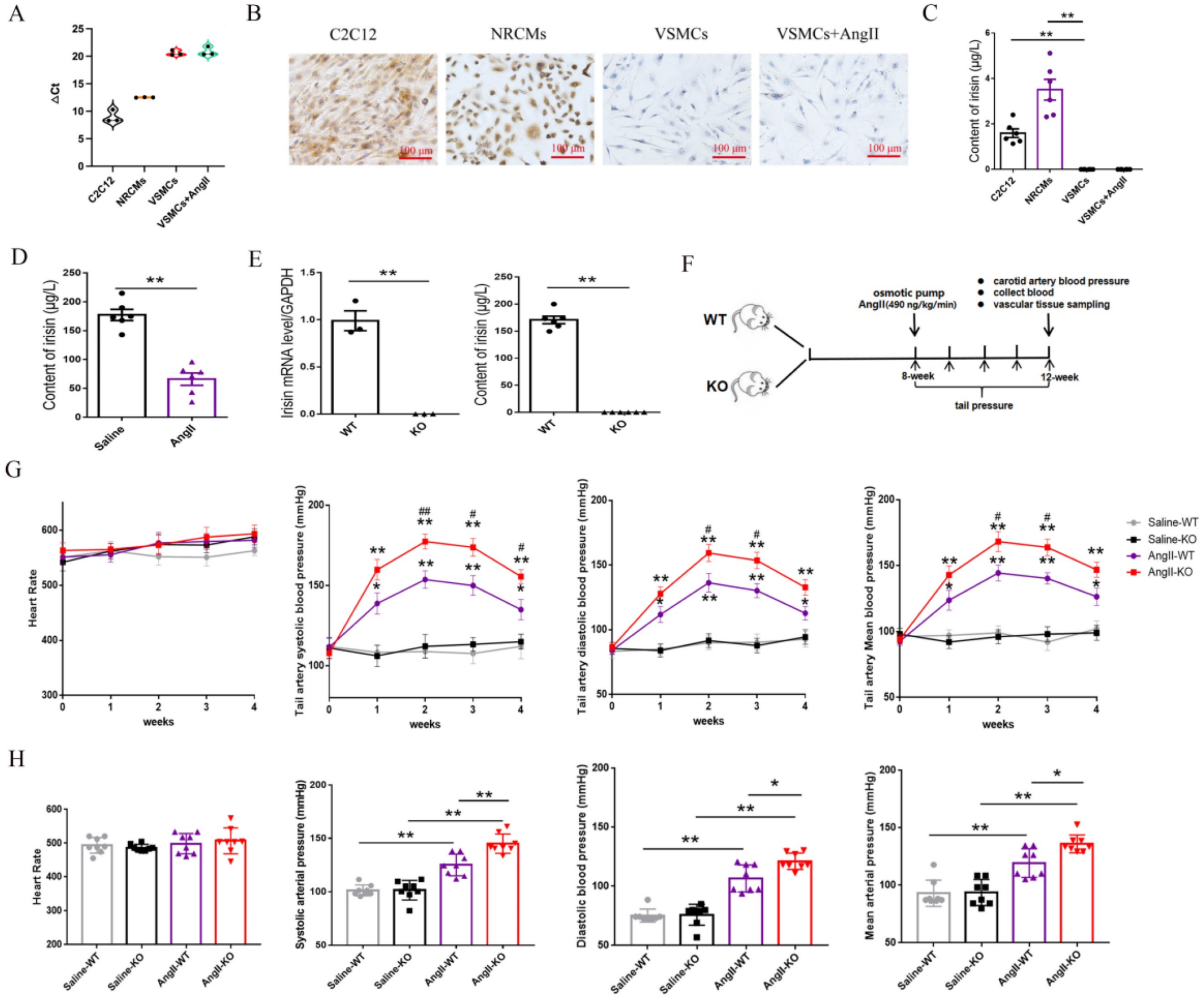
*FNDC5*/irisin expression in VSMCs, and the changes of blood pressure and heart rate in Ang II-treated hypertensive mice. A. The circle number for *FNDC5* gene amplification in real-time PCR assays in different cells, including C2C12 cells, NRCMs and mouse VSMCs. B. The immunohistochemical staining of *FNDC5* in C2C12 cells, NRCMs and mouse VSMCs. C. The immunoreactive irisin contents in cell culture supernatants of C2C12 cells, NRCMs and mouse VSMCs, respectively (n=6/group). D. Irisin contents of plasma in WT mice treated with saline or Ang II (490 ng/min/kg, 4 weeks) (n=6/group). E. The mRNA expression levels of *FNDC5* in aortic tissues in WT and irisin-KO mice (left panels, n=3/group), and irisin contents of plasma in WT mice and KO mice (right panels, n=6/group). F. Flow chart for hypertension experiment *in vivo*. G. The dynamic changes of heart rate and caudal artery blood pressure in WT mice and irisin-KO mice with Ang II (490 ng/min/kg) treatment for 4 weeks (n=8/group). *P<0.05, **P<0.01 vs the WT saline-treated mice; ^#^P<0.05, ^##^P<0.01 vs Ang II-treated WT mice. H. Effect of irisin deficiency on heart rate and blood pressure of common carotid artery after 4-week Ang II exposure (n=8/group). The data were presented as mean ± S.E.M. Paired *t*-test was used to analyze the data in D and E, while one-way ANOVA was used to analyze the data in C, G, and H. *P < 0.05 and **P < 0.01. WT, wild type mice; KO, *FNDC5* knockout mice; Ang II, angiotensin II.

Irisin mRNA expression was assessed using real-time PCR assays, while irisin protein expression was determined through immunohistochemical staining with a specific irisin antibody in VSMCs. As shown in Figure 1A, the △Ct value for irisin mRNA amplification was significantly higher in VSMCs (more than 20) compared to the myoblast cell line C2C12 cells (approximately 9) and neonatal rat cardiomyocytes (NRCMs, about 12). This suggests that the expression levels of irisin mRNA were much lower in VSMCs than in the control C2C12 cells and NRCMs. Additionally, the immunostaining of irisin was notably strong in both C2C12 cells and NRCMs, but almost absent in VSMCs (Fig.1B). Furthermore, an ELISA kit was utilized to measure irisin contents in the cell culture supernatants. The irisin contents were found to be 1.59 ± 0.19 μg/L and 3.51 ± 0.45 μg/L in the culture supernatants of C2C12 cells and NRCMs, respectively, but below the detection limit in VSMCs (Fig. 1C). Interestingly, the mRNA and protein expression of *FNDC5* in VSMCs, and the irisin contents in VSMC culture supernatants, showed no significant effect upon Ang II challenge.

### 3.2 Irisin gene deletion aggravated the increased blood pressure in Ang II-insulted hypertensive mice

C57BL/6 mice (8-week-old) were subcutaneously infused with Ang II in an osmotic pump for 4 weeks. The levels of irisin in plasma were significantly reduced compared to the control mice infused with saline (Fig. 1D), suggesting that the down-regulation of irisin may be involved in the development of hypertension. To examine the role of irisin in Ang II-induced hypertension, we studied the pathological changes caused by hypertension in irisin-KO mice. The irisin-KO mice showed normal appearance at birth with no signs of growth retardation or disorders. There was no mRNA expression of irisin in aortic tissues, and irisin immunoactivity in plasma was not detected (Fig. 1E). Caudal arterial pressure was measured weekly using a noninvasive tail-cuff system in 8-week-old WT or irisin-KO mice treated with Ang II for 4 weeks (Fig. 1F). There was no significant difference in SBP, MABP, and DBP between control irisin-KO and control WT mice at baseline and during 4-week saline infusion (Fig. 1G). However, the tail artery pressure of both WT and irisin-KO mice was increased at 1 week of Ang II exposure, and then gradually risen to reach a maximum at 2 weeks (Fig. 1G). Interestingly, Ang II-treated irisin-KO mice had a significant increase in SBP at 2, 3 and 4 weeks, MABP and DBP at 2 and 3 weeks, compared to Ang II-treated WT mice (Fig. 1G). This suggests that the deficiency of the irisin gene significantly exacerbated the increase in caudal arterial pressure in Ang II- treated WT mice.

At the end of the Ang II challenge, a cannulation procedure was performed to measure the carotid artery pressure in mice. The baseline carotid artery SBP, MABP, and DBP showed no significant difference between control WT mice (101.25±5.03, 93.07±11.29, and 75.35±5.49 mmHg) and control irisin-KO mice (101.41±9.06, 94.67±10.17, and 76.05±8.85 mmHg) without any treatment, respectively. The blood pressure in arteries of both WT and irisin-KO mice was significantly elevated by Ang II treatment. Furthermore, Ang II-treated irisin-KO mice had higher SBP, MABP, and DBP of the carotid artery than Ang II-insulted WT mice (Fig. 1H). Moreover, Ang II treatment did not cause any noticeable change in heart rates in either WT or irisin-KO mice, compared to their control counterparts, respectively (Fig. 1H).

### 3.3 Irisin gene deletion aggravated the aorta and mesenteric artery remodeling induced by Ang II exposure

**Figure 2:**
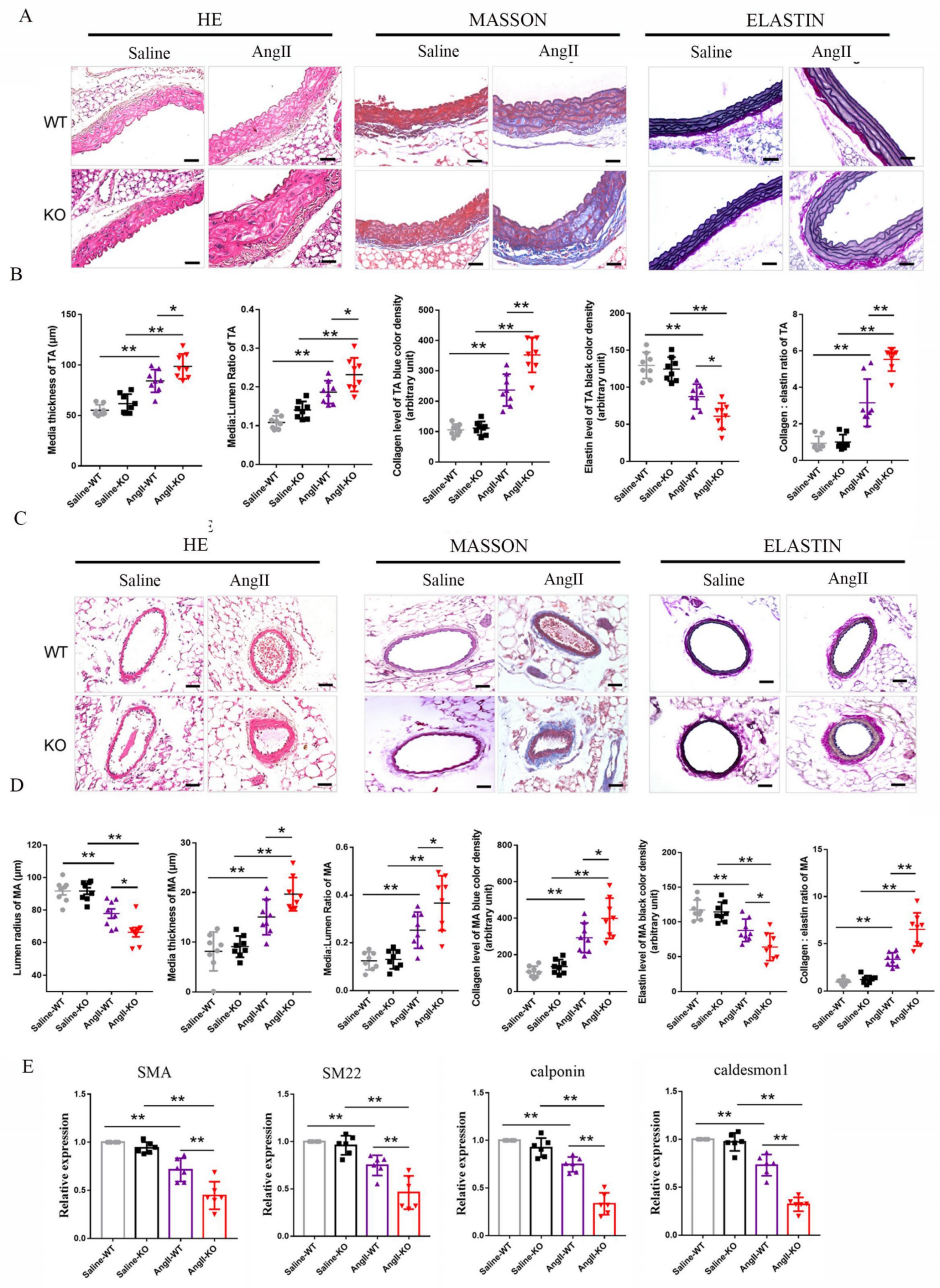
Irisin deficiency aggravated aorta and mesenteric artery remodeling in hypertensive mice insulted by Ang II exposure. A and B. Pathological vascular remodeling of thoracic aortas of irisin-KO and WT mice treated with saline or Ang II (490 ng/min/kg, 4-week) (n=8/group). H-E, Masson trichrome blue (rambling blue represents the deposition of collagen), and Elastin (blackish-brown represents elastin) staining of thoracic aorta vessel sections, respectively. Magnification 200×, scale bar: 50μm. C and D. Vascular remodeling of mesenteric artery sections in irisin-KO and WT mice (n=8/group). Magnification 200 ×, scale bar: 50μm. E. The mRNA expression levels of VSMC-specific contractile genes, including SMA, SM22, calponin, and caldesmon1, in thoracic aortic tissues obtained from irisin-KO and WT mice (n=6/group), respectively. (n=6/group). The data were presented as mean ± S.E.M., and the statistical analyses were performed by one way ANOVA. *p < 0.05 and **P < 0.01. WT, wild type mice; KO, *FNDC5* knockout mice; Ang II, angiotensin II.

Vascular remodeling is a major pathological change in hypertension, caused by persistent, chronic hemodynamic abnormalities in blood vessels 3. After analyzing histological staining of aortic tissues, we observed that Ang II challenge significantly increased the thickness of aortic media, the ratios of media/lumen, the deposition of collagen, and the ratios of collagen/elastin, while significantly decreasing the contents of elastin in the media of the aorta during the process of aortic remodeling in WT mice (Fig. 2A-2B). In irisin-KO mice, the Ang II challenge led to a larger increase in the thickness of aortic media, the ratios of media/lumen, the deposition of collagen, and the ratios of collagen/elastin, and a greater reduction in the contents of elastin in the aortic media compared to WT mice (Fig. 2A-2B). There was no significant change in the lumen radius between Ang II-treated irisin-KO, WT mice, and their saline-treated counterparts (Supplement Fig. 2).

Similarly, in mesenteric arteries, Ang II challenge significantly increased the thickness of vascular media, the ratios of media/lumen, the deposition of collagen, and the ratios of collagen/elastin, as well as significantly decreased the radius of lumen and the contents of elastin in the media (Fig. 2C-2D). Compared to Ang II-insulted WT mice, Ang II challenge induced greater the thickness of vascular media, the ratios of media/lumen, the deposition of collagen, and the ratios of collagen/elastin, while resulting in a smaller radius of lumen and lower expression of elastin in the mesenteric arteries of irisin-KO mice (Fig. 2C).

Phenotypic transformation in VSMCs is a critical cellular event in the process of hypertension remodeling 2, 3. We further detected the expression of contractile phenotype markers in aortic tissues, including SMA, smooth muscle protein of 22 kDa (SM22), calponin, and caldesmon 1. There was no significant difference in baseline mRNA levels of contractile phenotype genes between WT and irisin-KO mice. The expression of contractile phenotype gene mRNAs (including SMA, SM22, calponin, and caldesmon 1) in the aortic tissue of Ang II-insulted WT mice was significantly reduced compared to control WT mice. This reduction was further significantly exacerbated by irisin deficiency (Fig. 2E).

### 3.4 FNDC5 overexpression attenuated the increased blood pressure and vascular remodeling in Ang II-insulted hypertensive mice

**Figure 3:**
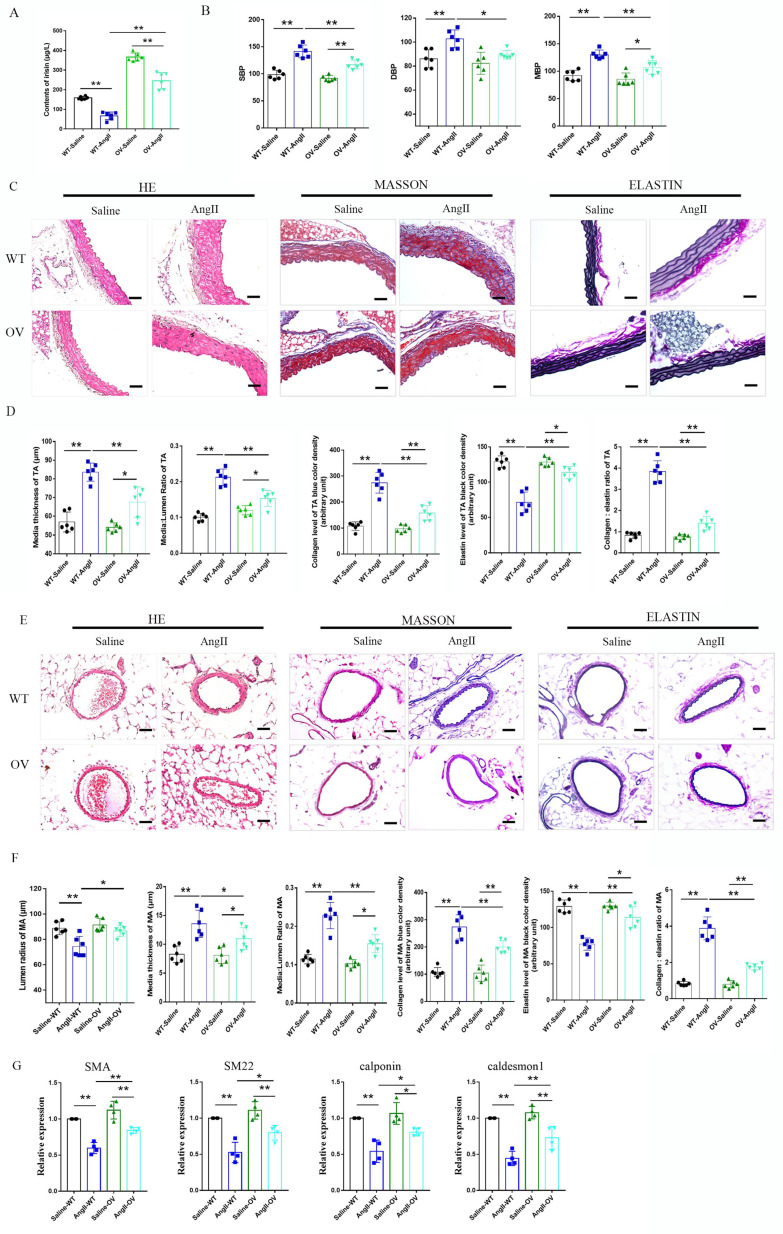
Irisin gene overexpression attenuated blood pressure and vascular remodeling in Ang II-treated hypertensive mice. A. Irisin contents in plasma of WT mice and *FNDC5* gene overexpression (OV) mice in the absence and the presence of Ang II (490 ng/min/kg) exposure for 4 weeks (n=6/group). B. Effect of *FNDC5* gene overexpression on blood pressure of common carotid artery after 4-week Ang II exposure (n=8/group). C-F. The analysis of vascular remodeling in thoracic aorta and mesenteric artery sections from WT and OV mice treated with saline or Ang II (490 ng/min/kg, 4 weeks, n=6/group). H-E, Masson trichrome blue (rambling blue represents the deposition of collagen), and Elastin (blackish-brown represents elastin contents) staining of vascular sections, respectively. Magnification 200 ×, scale bar: 50μm. G. The mRNA expression levels of VSMC-specific contractile genes (including SMA, SM22, calponin, and caldesmon1) in aortic tissues of WT and OV mice treated with saline or Ang II (490 ng/min/kg, 4 weeks), respectively (n=4/group). The data were presented as mean ± S.E.M. Paired *t*-test was used to analyze the data in A, while one-way ANOVA was used to analyze the data in B, D, F, and G. *P < 0.05 and **P < 0.01. WT, wild type mice; OV, *FNDC5* overexpression mice; Ang II, angiotensin II; SBP, systolic blood pressure; MBP, mean arterial blood pressure; DBP, diastolic blood pressure.

The role of irisin playing in hypertensive vascular remodeling was further investigated in *FNDC5* overexpression (OV) mice. As shown in Fig. 3A, the irisin contents in the plasma of OV mice were significantly higher than that of WT mice, which was significantly decreased by the Ang II challenge. However, the plasma irisin content in Ang II-treated OV mice was significantly higher than that in Ang II-treated WT mice. No significant difference was observed in blood pressure between WT and OV mice at baseline. After four weeks of Ang II treatment with osmotic pumps, both WT and OV mice showed an apparent increase in carotid blood pressure. However, the blood pressure of OV mice exposed to Ang II was much lower than that of WT mice with Ang II treatment (Fig. 3B). *FNDC5* overexpression significantly reduced vascular remodeling in Ang II-treated WT mice, by decreasing the thickness of media, the ratios of media/lumen, the deposition of collagen, and the ratios of collagen to elastin, while increasing the contents of elastin in the media of both aortas and mesenteric arteries, and mesenteric artery's lumen radius, compared to Ang II-insulted WT mice (Fig. 3C-3F). In aortic tissue, *FNDC5* overexpression also significantly attenuated the suppression of Ang II on the expression of contraction genes such as SMA, SM22, calponin, and caldesmon 1 (Fig. 3G).

### 3.5 FNDC5 deletion aggravated and its overexpression attenuated VSMC proliferation in Ang II-insulted aorta

**Figure 4:**
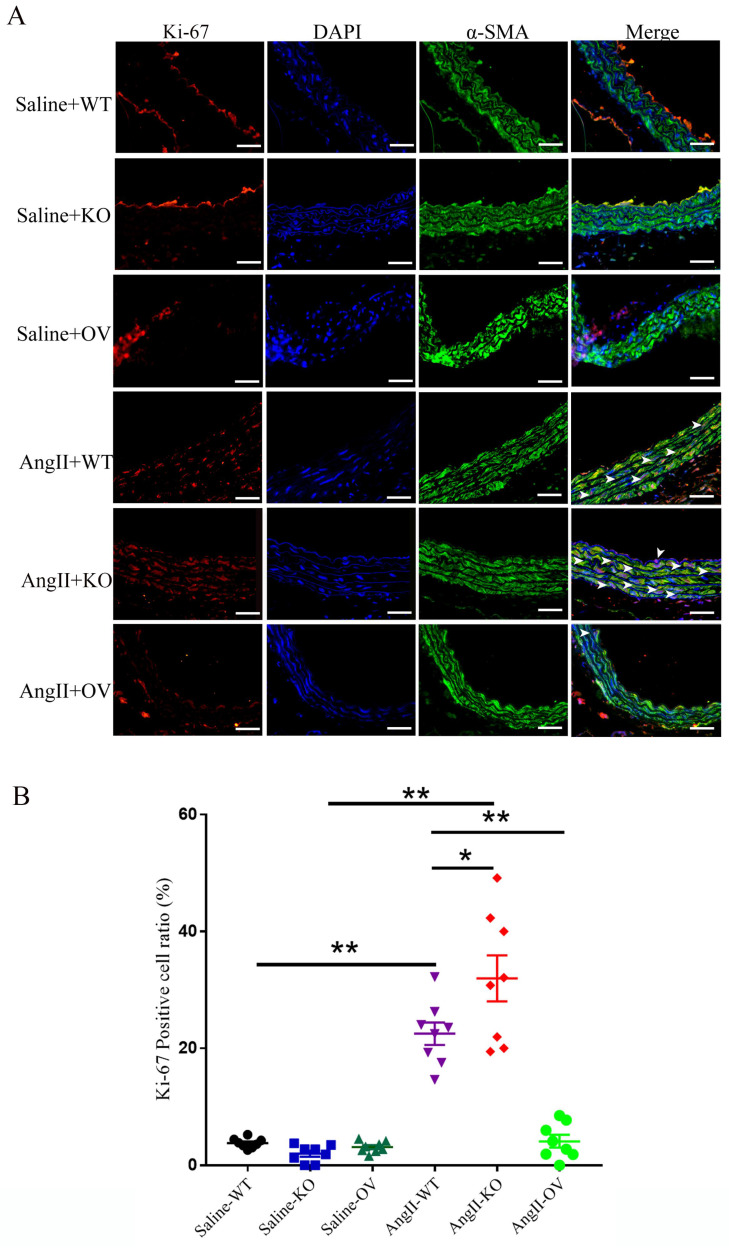
The influence of irisin on VSMC proliferation in aortic media in Ang II-induced hypertensive mice. A. Representative images of Ki-67 immunostaining (red) in aortic sections. VSMCs were identified by α-smooth muscle actin staining (green), and nuclei were marked by DAPI staining (blue). The white arrows indicated Ki-67-positive VSMCs (rose red). Magnification 200×, scale bar =50 μm. B. The percentage of Ki-67-positive VSMCs represented by the ratios of Ki-67-positive VSMCs to the total VSMCs in the aortic media (n = 8/group). Data are mean ± S.E.M., and the statistical analyses were performed by one way ANOVA. *P < 0.05 and **P < 0.01. WT, wild-type mice; KO, *FNDC5* knockout mice; OV, *FNDC5* overexpression mice; Ang II, angiotensin II.

Ki-67, a widely used indicator for cellular proliferation, is found in the nuclei and is strictly associated with VSMC proliferation and growth 30. As shown in Fig. 4, low levels of VSMC Ki-67 labeling indexes were found in the media of thoracic aortas of control WT, irisin-KO, and irisin-OV mice with saline treatment, with no significant difference between them. These three different genotypes of mice showed a noticeable increase in VSMC Ki-67 labeling indexes in the aortic media due to the Ang II challenge, compared to the control counterparts. Compared to control WT mice, Ang II treatment induced a 4. 9-fold increase (P < 0. 01) in Ki-67 positive VSMCs in the media of aortas of WT mice. This increase was significantly enhanced by irisin deficiency by 42% (P < 0. 05), while significantly reduced by *FNDC5* overexpression by 81% (P < 0. 01), compared to WT mice with Ang II alone treatment (Fig. 4B).

### 3.6 Irisin supplementation suppressed the proliferation, migration, and phenotypic transformation of VSMCs challenged by Ang II

**Figure 5:**
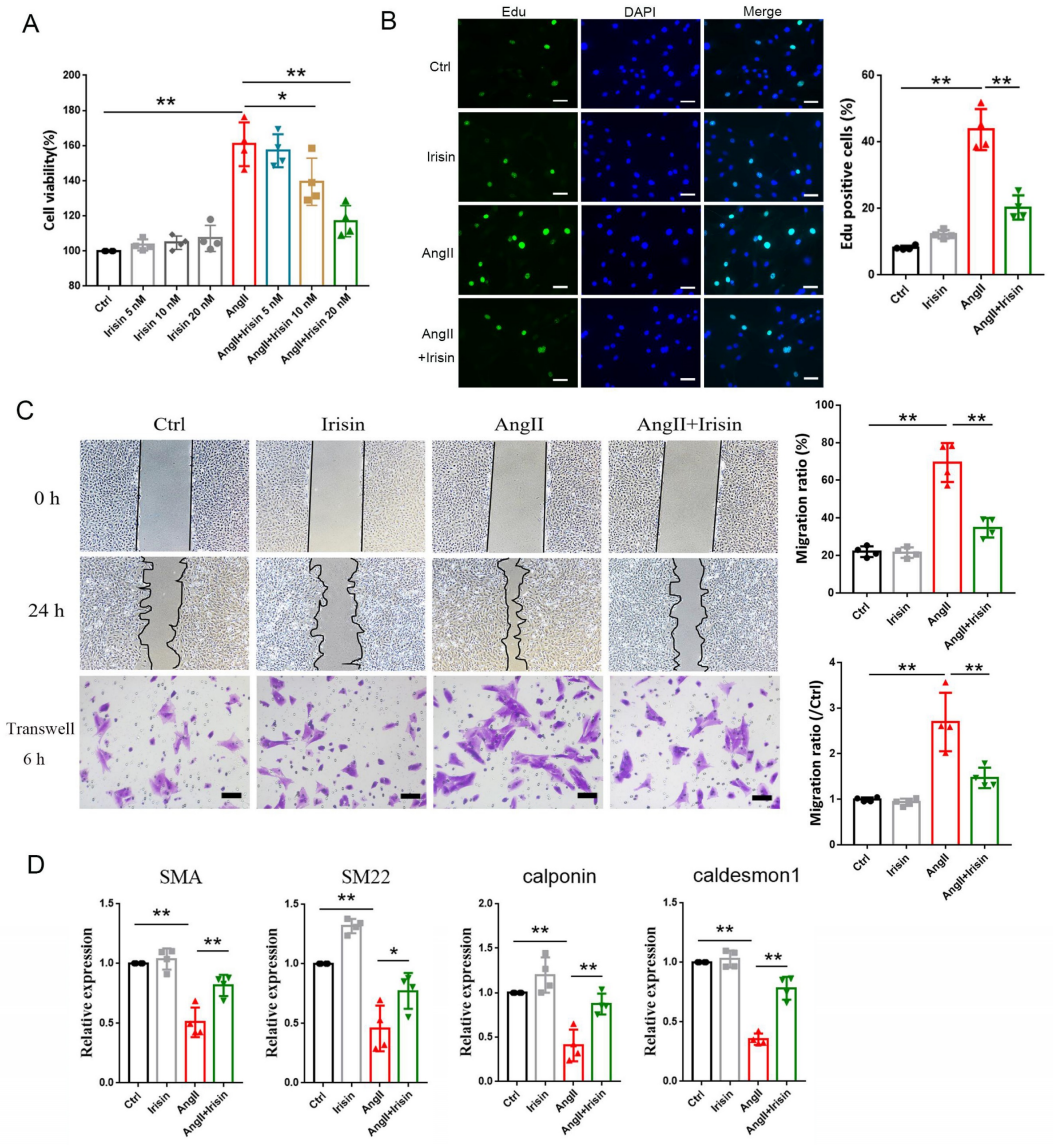
Irisin supplementation suppressed the proliferation, migration and phenotypic transformation in Ang II-challenged VSMCs. A. The cell viability in cultured primary mouse VSMCs challenged by Ang II (1 μM) in the absence and presence of exogenous irisin (5, 10, 20 nM) co-incubation for 24 h (n=4/group). B. Edu incorporation assays evaluated the proliferation ability of VSMCs challenged by Ang II (1 μM) in the absence and presence of exogenous irisin (20 nM) co-incubation for 24 h (n=4/group). C. Scratch and transwell migration assays evaluated the migration of VSMCs challenged by Ang II (1 μM) in the absence and presence of exogenous irisin (20 nM) co-incubation for 24 h (n=4/group). D. Phenotypic switching indicated by the mRNA expressions of VSMC-specific contractile genes (including SMA, SM22, calponin, and caldesmon1) in VSMCs challenged by Ang II (1 μM) in the absence and presence of exogenous irisin (20 nM) co-incubation for 24 h (n=5/group). The data were presented as mean ± S.E.M., and the statistical analyses were performed by one way ANOVA. *P < 0.05 and **P < 0.01. Ctrl, control; Ang II, angiotensin II; SMA, SM α-actin; SM22, smooth muscle 22 alpha.

VSMCs, the most abundant cells in vessel walls, play an essential role in hypertensive progression 2. In hypertension, vascular remodeling is characterized by VSMC proliferation, which leads to an increase in wall mass and narrowing of the lumen 21, 23. The viabilities of VSMCs were significantly improved by 1 μM Ang II exposure compared to the control cells, but this improvement was significantly blunted in a concentration-dependent way by irisin supplementation (Fig. 5A). Additionally, Ang II treatment led to a significant increase in Edu incorporation in VSMCs, which was significantly decreased by 54. 5% with 20 nM irisin supplementation (Fig. 5B).

VSMC migration is important in the pathophysiology of hypertension, and scratch assays as well as transwell assays were preformed to identify the role of irisin in VSMC migration induced by Ang II. VSMC migration in both assays was significantly promoted by Ang II exposure alone compared to control VSMCs. However, irisin supplementation significantly attenuated Ang II-induced VSMC migration compared to Ang II alone-treated cells (Fig. 5C).

The phenotypic transformation of VSMCs from a contractile phenotype into a synthetic phenotype, is a critical initiator for hypertensive vascular remodeling. To assess the function of irisin in Ang II-regulated VSMC phenotypes, we examined the specific contractile phenotype markers of VSMCs. In Fig. 5D, we observed that irisin alone-challenge led to a noticeable increase in mRNA levels of SM22 in VSMCs compared to the non-processed control cells. The mRNA expressions of markers for VSMC contractile phenotype, such as SMA, SM22, calponin, and caldesmon 1, were drastically reduced by Ang II alone-treatment, but were clearly reversed by exogenous irisin supplementation.

### 3.7 FNDC5 deficiency aggravated and its overexpression attenuated Ang II-induced disruption of intracellular calcium homeostasis and ER stress in the aortic tissues of hypertensive mice

**Figure 6:**
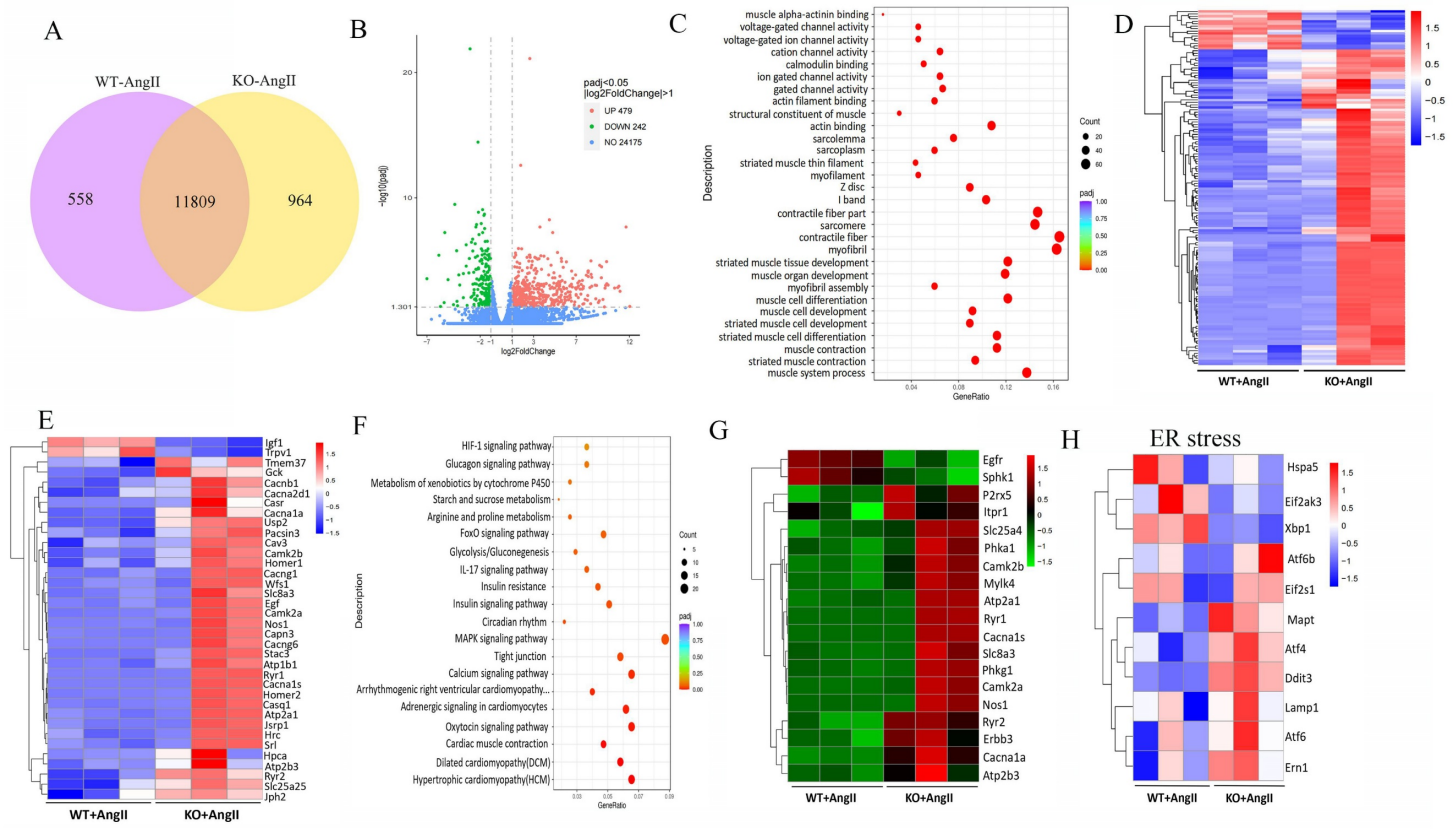
*FNDC5* deficiency disrupted intracellular calcium homeostasis and induced ER stress in aortic tissues of AngII-treated hypertensive mice. A. Venn diagram showed the number of uniquely expressed genes in aortic tissues of WT and KO mice with Ang II treatment. B. The volcano map visualized the distribution of differential genes (including up-regulation and down-regulation) in the aortic tissues of WT and KO mice treated with Ang II (490 ng/min/kg) for 4 weeks. C. RNA-seq GO enrichment analyses of gene expression (top 30) in aortic tissues of WT and KO mice after Ang II (490 ng/min/kg) exposure for 4 weeks. D. RNA-seq heat map compared the top 30 clustering features of GO enrichment in aortic tissues of WT and KO mice after Ang II (490 ng/min/kg) exposure for 4 weeks. Each column represents an individual mouse. E. The heat map of calcium signaling pathway from GO enrichment in aortic tissues of WT and KO mice after Ang II (490 ng/min/kg) exposure for 4 weeks. F. RNA-seq KEGG pathway enrichment analyses of gene expression in aortic tissues of WT and KO mice challenged with Ang II (490 ng/min/kg) for 4 weeks. G. The heat map of calcium signaling pathway from KEGG pathway enrichment in aortic tissues of WT and KO mice challenged with Ang II (490 ng/min/kg) for 4 weeks. H. The heat map of expression of ER stress-related genes in aortic tissues of WT and KO mice after Ang II (490 ng/min/kg) exposure for 4 weeks.

**Figure 7:**
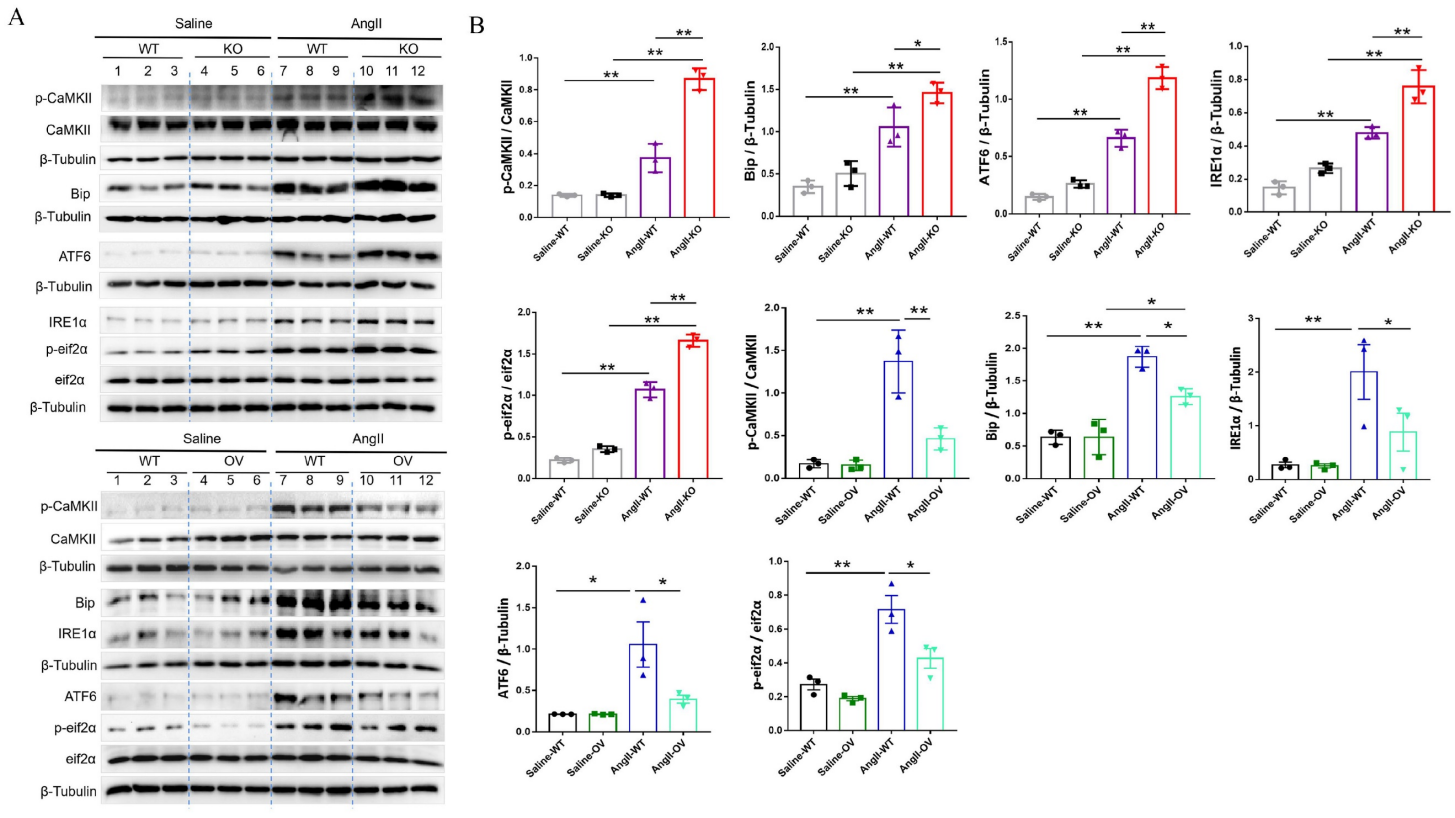
*FNDC5* deficiency exacerbated, and its overexpression alleviated the intracellular calcium imbalance and ER stress in Ang II-insulted mouse aortic tissues. A. Lysates from aortic tissues of WT, irisin-KO and irisin-OV mice infused with saline or Ang II (490 ng/min/kg) for 4 weeks were analyzed by immunoblotting. The expression levels of calcium-signaling related proteins (including p-CaMK II, and CaMK II) and ER stress-related proteins (including Bip, ATF6, IRE1α, p-eif2α, and eif2α) were detected by using specific antibodies, respectively. B. Quantitative measurement (n=3/group). The data were presented as mean ± S.E.M., and the statistical analyses were performed by one way ANOVA. *P < 0.05 and **P < 0.01. WT, wild type mice; KO, irisin knockout mice; OV, irisin overexpression mice; Ang II, angiotensin II.

To further determine the potential molecular mechanisms of irisin involved in its protection against hypertensive vascular remodeling, the gene expression profiles of aortic tissues in Ang II-infused WT and irisin-KO mice were analyzed by RNA-seq. Venn map (Fig. 6A), volcano map (Fig. 6B), and cluster maps (Fig. 6C) were drawn based on the differentially expressed transcripts (DETs). In the aortic tissues of Ang II-insulted irisin-KO mice, 479 transcripts were upregulated, and 242 transcripts were downregulated compared to Ang II-challenged WT mice (Fig. 6B). The differentially expressed genes were mainly concentrated in muscle development, muscle contraction, and ion channel activity, with most of them being significantly up-regulated according to GO enrichment analyses (Fig. 6C, 6D). The mRNA levels of genes involved in the calcium signal pathway in GO enrichment analyses showed significant changes (Fig. 6E). KEGG enrichment analyses of signaling pathways revealed that calcium signaling pathways related to ion channel activity were significantly upregulated (Fig. 6F), and the heat maps showed the mRNA expression of genes associated with intracellular calcium signaling pathways, including RYR2, IP3R, CaMK II, Slc8a3, Cacna1s, and Atp2a1, was significantly increased, (Fig. 6G). The calcium homeostasis in the endoplasmic reticulum (ER) serves as a major player in maintaining intracellular calcium signaling. Alterations in ER Ca^2+^ homeostasis lead to unfolded protein accumulation, causing ER stress and subsequently triggering the unfolded protein response 27. The analyses of mRNA expression of ER stress-related genes showed a significant increase in ATF6, Eif2s1, ATF4, Ddit3, Lamp1, and Ern1 gene expression, as well as a significant decrease in Hspa5, Eif2ak3, and Xbp1 gene expression (Fig. 6H). Ang II infusion induced calcium homeostasis imbalance and severe ER stress in mouse aortic tissues. We further investigated the regulatory effect of irisin on key protein expressions of the calcium signaling pathway and the endoplasmic reticulum stress pathway. The protein levels of Ca^2+^ homeostasis- and ER stress-associated proteins, including phosphorylated-CaMK II, Bip, IRE1α, ATF6, and phosphorylated-eif2α, were low at baseline in the aortic tissues of WT, irisin-KO, and irisin-OV mice (Fig. 7A-7B). Ang II challenge significantly hooted the protein levels of phosphorylated-CaMK II and the four ER stress-related proteins in the aortic tissues in WT and KO mice. Furthermore, the levels of all these five proteins in the aortic tissues were higher in Ang II-insulted KO mice and lower in OV mice exposed to Ang II compared to WT mice with Ang II alone challenge.

### 3.8 Irisin supplementation inhibited intracellular calcium imbalance-mediated ER stress induced by Ang II exposure

**Figure 8:**
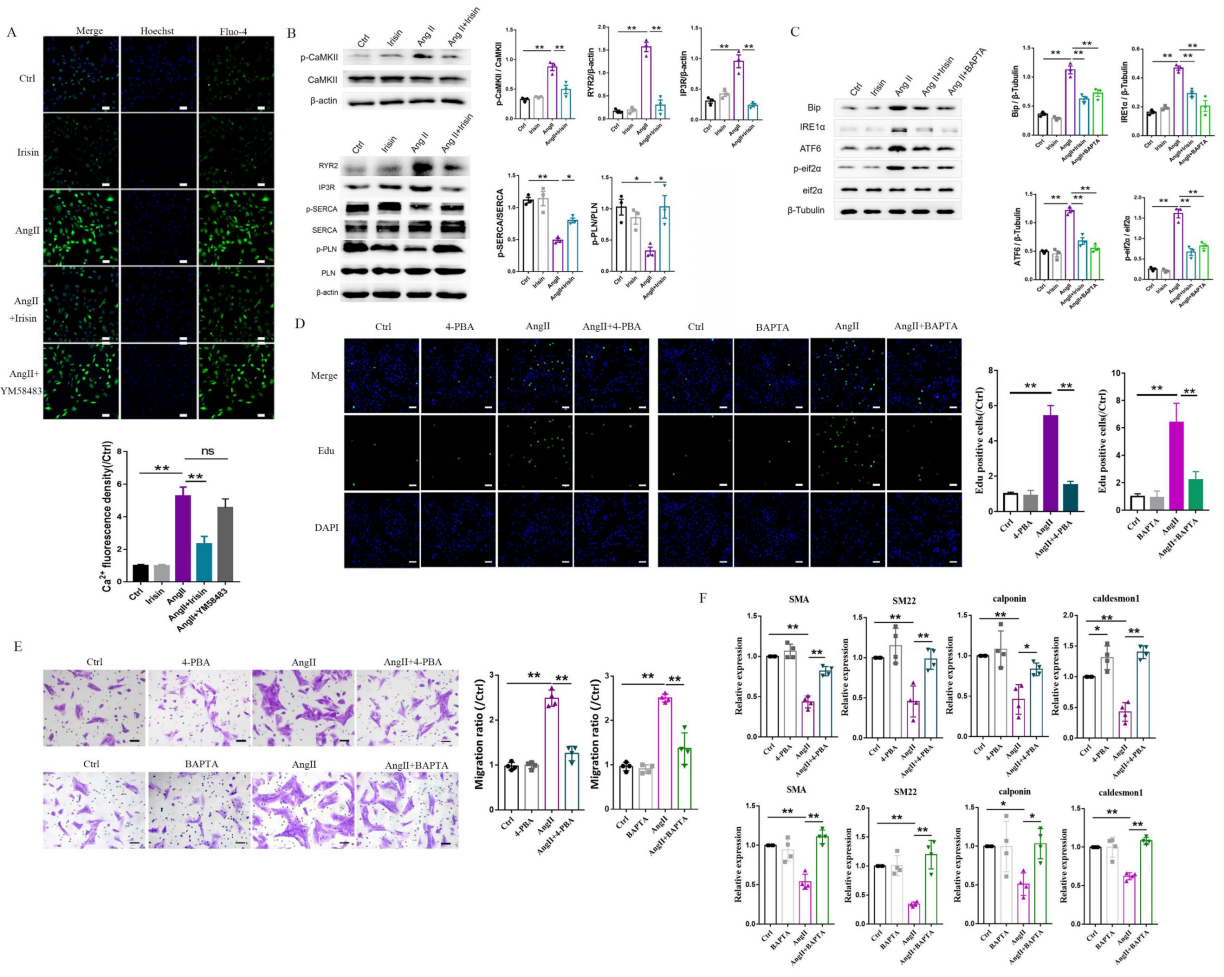
Irisin supplementation alleviated intracellular calcium imbalance-mediated ER stress induced by Ang II exposure. A. Ca^2+^ fluorescent probe assays evaluated intracellular calcium levels in VSMCs challenged with Ang II (1 μM) or irisin (20 nM) alone, or irisin (20 nM) or YM-58483 (a SOCE inhibitor, 10 μM) pre-treated for 30 min, and then co-incubation with Ang Ⅱ (1 μM) for another 6 h (n=4/group). B. The effect of irisin supplementation (20 nM) on the expression levels of calcium-signaling related proteins in VSMCs with Ang II exposure (1 μM, n=3/group). C. The effect of irisin (20 nM) or BAPTA-AM (a Ca^2+^ chelator, 1 μM) supplementation on the expression of ER stress-related proteins in VSMCs with Ang II (1 μM) exposure (n=3/group). D. Edu incorporation assays evaluated the proliferation ability of VSMCs treated with Ang II (1 μM), or 4-PBA (an ERS inhibitor, 1mM), or BAPTA-AM (a Ca^2+^ chelator, 1 μM) alone, or Ang II (1 μM) in combination with 4-PBA (1mM) or BAPTA-AM (1 μM) for 24 h, respectively (n=4/group). E. Transwell migration assays evaluated the migration of VSMCs treated with Ang II (1 μM), or 4-PBA (an ERS inhibitor, 1mM), or BAPTA-AM (a Ca^2+^ chelator, 1 μM) alone, or Ang II (1 μM) in combination with 4-PBA (1mM) or BAPTA-AM (1 μM) for 24 h, respectively (n=4/group). F. Phenotypic switching indicated by the mRNA expressions of VSMC-specific contractile-genes (including SMA, SM22, calponin, and caldesmon1) in VSMCs treated with Ang II (1 μM), or 4-PBA (an ERS inhibitor, 1mM), or BAPTA-AM (a Ca^2+^ chelator, 1 μM) alone, or Ang II (1 μM) in combination with 4-PBA (1mM) or BAPTA-AM (1 μM) for 24 h, respectively (n=4/group). The data were presented as mean ± S.E.M., and the statistical analyses were performed by one way ANOVA. *P < 0.05 and **P < 0.01. Ctrl, control; Ang II, angiotensin II; SMA, SM α-actin; SM22, smooth muscle 22 alpha.

Based on RNA-seq results from hypertensive remodeling aortic tissues, the regulation of irisin on Ang II-induced Ca^2+^ signaling pathways were further examined in cultured primary VSMCs. Intracellular calcium levels in VSMCs were significantly increased by Ang II stimulation, observed through Ca^2+^ fluorescent probe staining (Fig. 8A). The plasma membrane calcium-permeable channels and the endoplasmic reticulum (calcium reservoirs)-mediated release of calcium cause the influx of extracellular calcium, this then leads to increased intracellular calcium levels 27,34. However, the supplementation of YM-58483 (10 µmol/L), a blocker of store-operated calcium entry (SOCE), partially reduced the increased intracellular calcium levels caused by Ang II exposure with no statistical significance, suggesting that the increase in intracellular calcium induced by Ang II might be mainly derived from calcium release from the ER. Irisin supplementation significantly decreased intracellular calcium levels increased by Ang II exposure (Fig. 8A). Calmodulin-dependent protein kinase II (CaM II), a highly conserved serine/threonine specific protein kinase, is modulated by the Ca^2+^/ calmodulin complex and is critical in maintaining VSMC calcium homeostasis and reuptake 35. Ang II exposure significantly increased phosphorylation of CaMK II in VSMCs, while irisin pre- and co-incubation significantly reduced Ang II-induced phosphorylation of CaMK II (Fig. 8B), indicating that irisin negatively regulates excessive intracellular calcium. These results are consistent with those obtained from RNA-seq assays in aortic tissues. As key regulators of intracellular calcium homeostasis, RyR2 and IP3R are located in the membrane of ER and serve as major calcium release channels. The sarcoplasmic reticulum calcium-ATPase (SERCA) pump transports Ca^2+^ from the cytosol back to the ER, and it is further regulated by phospholamban (PLN) 22. Ang II exposure up-regulated RYR2 and IP3R protein levels and down-regulated the phosphorylation levels of SERCA and PLN, which were reversed by irisin supplementation (Fig. 8B). Disruption of calcium signaling due to cellular dyshomeostasis induces an ER stress response 36. Further investigation was conducted to explore the effects of irisin on ER stress in Ang II-treated VSMCs. The expression levels of ER stress-associated proteins, including Bip, IRE1α, ATF6, and p-eif2α, in VSMCs were significantly increased due to Ang II exposure (Fig. 8C). Supplementation of irisin significantly decreased their levels in Ang II-challenged VSMCs, which was comparable to the reduction in expression levels of ER stress-associated proteins resulting from Ang II exposure when treated with BAPTA-AM, a calcium scavenger. Furthermore, we observed that supplementation with BAPTA-AM or 4-PBA, an ERS inhibitor, significantly inhibited VSMC proliferation, migration, and phenotypic transformation induced by Ang II (Fig. 8D-8F).

### 3.9 Irisin suppressed ER stress to attenuate Ang II-induced VSMC remodeling via the αV/β5 receptor

**Figure 9:**
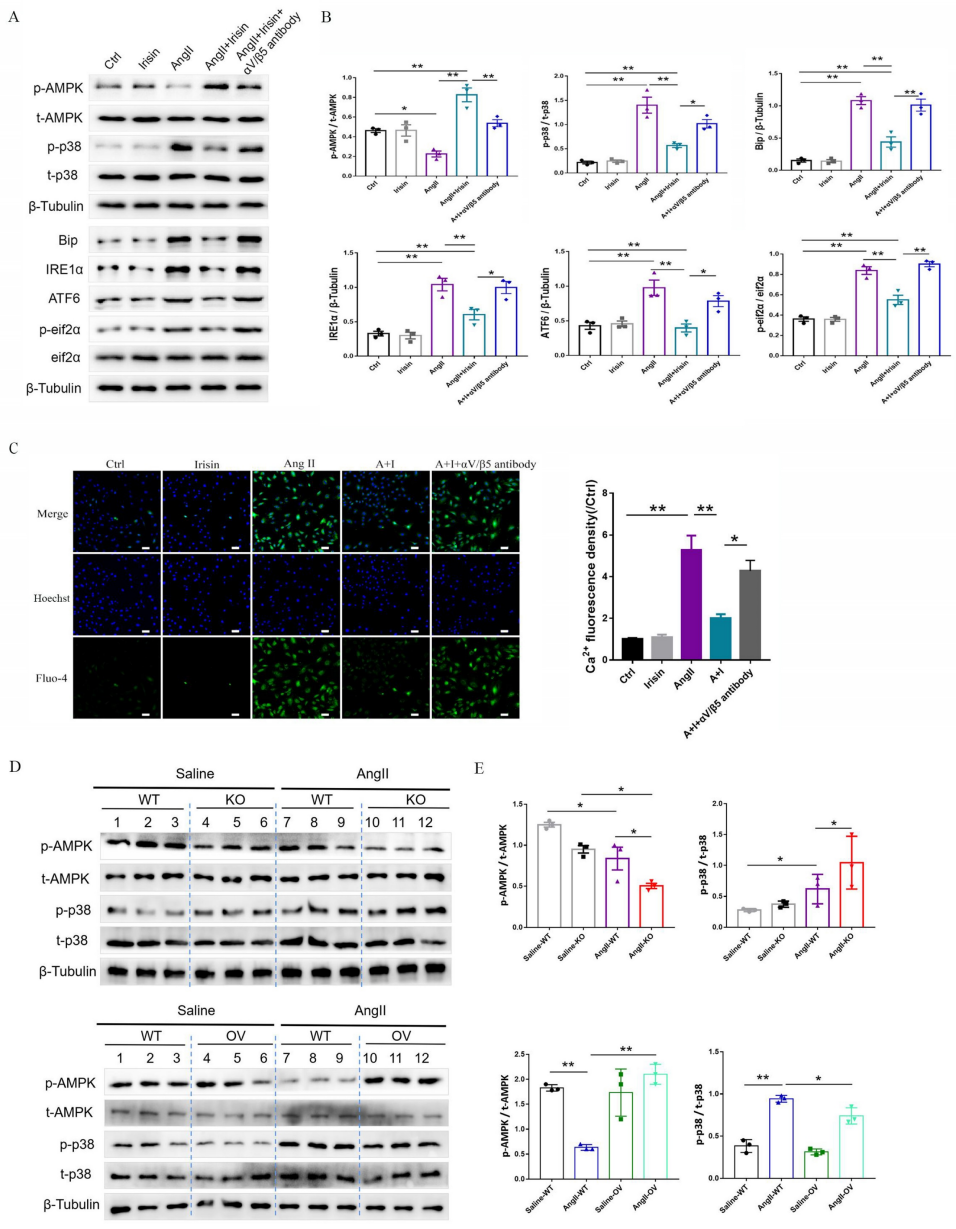

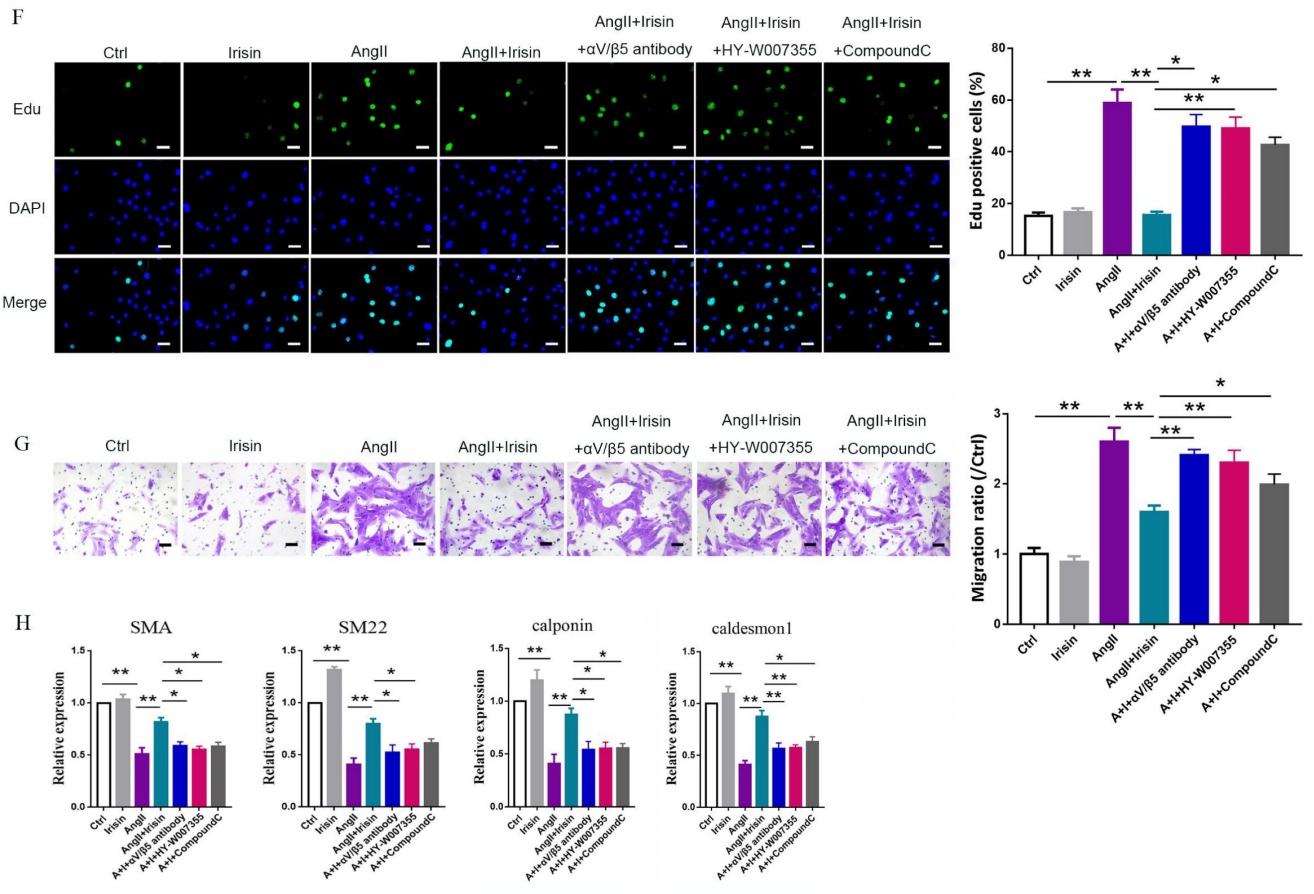
Irisin inhibited calcium overload, ER stress and remodeling in VSMCs and aortic tissues with Ang II exposure via αV/β5 receptor-AMPK/p38 pathway. A and B. The effect of irisin supplementation on the expression levels of ERS-related proteins and kinases (AMPK and p38) in Ang II-treated VSMCs was evaluated by immunoblotting. VSMCs were treated with irisin (20 nM) or Ang II (1 μM) alone, or irisin (20 nM) in combination with Ang II (1 μM) in the absence and presence of αV/β5 antibody (n=3/group). C. Intracellular calcium levels in VSMCs treated with irisin (20 nM) or Ang II (1 μM) alone, or irisin (20 nM) in combination with Ang II (1 μM) in the absence and presence of αV/β5 antibody (n=3/group). D-E. The levels of phosphorylated- and total-AMPK and p38 in lysates of aortic tissues from WT, irisin-KO, and irisin-OV mice infused with saline or Ang II (490 ng/min/kg) for 4 weeks were detected using immunoblotting (n=3/group). F. Edu incorporation assay evaluated the proliferation ability of VSMCs treated with irisin (20 nM) or Ang II (1 μM) alone, or irisin (20 nM) in combination with Ang II (1 μM) in the absence and presence of αV/β5 antibody, or compound C (an AMPK inhibitor, 5 μM), or HY-W007355 (a p38 activator, 10 μM) for 24 h (n=4/group). G. Transwell migration assay evaluated the migration of VSMCs treated with irisin (20 nM) or Ang II (1 μM) alone, or irisin (20 nM) in combination with Ang II (1 μM) in the absence and presence of αV/β5 antibody, or compound C (an AMPK inhibitor, 5 μM), or HY-W007355 (a p38 activator, 10 μM) for 24 h (n=4/group). H. Phenotypic switching indicated by the mRNA expressions of VSMC-specific contractile genes (including SMA, SM22, calponin, and caldesmon1) in VSMCs treated with irisin (20 nM) or Ang II (1 μM) alone, or irisin (20 nM) in combination with Ang II (1 μM) in the absence and presence of αV/β5 antibody, or compound C (an AMPK inhibitor, 5 μM), or HY-W007355 (a p38 activator, 10 μM) for 24 h (n=4/group). The data were presented as mean ± S.E.M., and the statistical analyses were performed by one way ANOVA. *P < 0.05 and **P < 0.01. Ctrl, control; I, irisin; Ang II, angiotensin II; SMA, SM α-actin; SM22, smooth muscle 22 alpha; WT, wild type mice; KO, irisin knockout mice; OV, irisin overexpression mice.

Integrin αV/β5 has been identified as one of the irisin receptors. In VSMCs, the mRNA expression levels of αV/β5 were not affected by Ang II or irisin treatment alone or in combination (Supplement Fig. 3A). However, supplementation of the αV/β5 antibody almost abrogated the inhibition of irisin on ER stress-related proteins induced by Ang II exposure, such as Bip, IRE1α, ATF6, and phosphorylated eif2α, and eliminated the inhibition of irisin on intracellular calcium overload in Ang II-treated VSMCs (Fig. 9A-9C).

Irisin transduces its cellular actions through Ser/Thr protein kinases, including AMPK, PI3K-Akt, and MAPKs 15, 16, 37. Ang II exposure induced a higher level of phosphorylation in AKT, p38, ERK, and JNK, and lower level of AMPK phosphorylation in VSMCs (Fig. 9A-9B and Supplement Fig. 3B-C). Irisin supplementation didn't change the activities of ERK, JNK, or AKT in Ang II-challenged VSMCs. However, it significantly increased AMPK phosphorylation levels and reduced p38 phosphorylation levels (Fig. 9A, 9B and Supplement Fig. 3B-C). In Ang II-insulted mouse aortic tissues, irisin deficiency resulted in lower AMPK phosphorylation and higher p38 phosphorylation compared to Ang II-treated WT mice, while irisin overexpression significantly reversed these Ang II-induced changes in WT mice. These *in vivo* results were consistent with the results obtained from Ang II-challenged VSMCs (Fig. 9D, 9E). Furthermore, pre- and co-incubation of VSMCs with the αV/β5 antibody, an AMPK inhibitor compound C, or a p38-MAPK activator HY-W007355 significantly reversed the suppression of irisin on Ang II-evoked proliferation, migration, and phenotypic transformation in VSMCs (Fig. 9F-9H).

## 4. Discussion

Vascular remodeling is an adaptive change in the function and structure of arteries in response to long-term hypertensive stimulation. It is an important pathological change and the structural basis for the maintenance and worsen of hypertensive diseases^3^. Irisin is a novel secreted myokine encoded by the *FNDC5* precursor gene and highly expressed in the heart, brain, liver, and skeletal muscle^9^. We found little mRNA and almost no protein expression of irisin in VSMCs, which is consistent with publicly available data from the Human Protein Atlas (Supplement Fig. 4A-B). Irisin was shown to be down-regulated in the plasma of mice exposed to Ang II, which is comparable with previous findings of Zhou et al.'s that mouse vascular tissues insulted with Ang II showed a significant decrease in both irisin mRNA and protein expression levels 38. Irisin acts in an autocrine/paracrine way in local tissues and mediates peripheral activity in a hormone-like manner when released into the circulation^7^, ^9^. Exogenous irisin had an action to reduce blood pressure levels in both healthy and hypertensive rats when administered intravenously. Additionally, irisin promotes mesenteric artery ring dilatation through ATP-sensitive potassium channels^12^. Our findings suggest that irisin secreted from other tissues and organs into the circulation may perform hormone-like effects on vascular media since there is almost no irisin protein expression in VSMCs. Decreased plasma levels of irisin may also contribute to the promotion of blood pressure levels in hypertensive diseases due to a lack of vasorelaxing agents 12, 17.

Persistent hypertension leads to increased arterial wall tension, triggering vascular remodeling, fibrosis, and stiffness 2. This is characterized by increased collagen deposition, reduced elastin content, and infiltration of pro-inflammatory cells 3. Our investigation provides definitive evidence that irisin is essential for VSMC ER activity and calcium homeostasis, playing a crucial role in controlling hypertension and vascular remodeling. We observed that Ang II challenge induced typical vascular remodeling phenotypes, including increased arterial blood pressure, elastin fragmentation, collagen deposition, VSMC proliferation, and changes in arterial morphology. These effects were significantly aggravated by *FNDC5* deficiency and attenuated by its overexpression. *In vitro* experiments using cultured VSMCs further confirmed these findings, showing that exogenous irisin supplementation substantially inhibited VSMC proliferation, migration, and phenotypic alteration induced by Ang II. These findings provide compelling evidence that irisin, similar to other vascular active factors such as vascular endothelial growth factor 39, ANP 40, and adiponectin^41^, is an essential regulator in hypertension-induced vascular remodeling.

The diameter of the vascular lumen and blood flow are primarily determined by VSMCs 3. Controlling the contractility of vascular smooth muscle depends heavily on dynamic changes in intracellular calcium^21^. Therefore, intracellular calcium is essential in controlling vascular functions, with physiological and pathological consequences. The inflow of Ca^2+^ through plasma membrane ion-permeable channels and the Ca^2+^ release from Ca^2+^ stores in cells, like the ER, are two sources of Ca^2+^ that can be employed for cytoplasmic signaling in VSMCs^27^. The multifunctional hormone Ang II activates a variety of intricate intracellular signaling pathways through its interaction with the AT1 receptor. Ang II triggers a cytosolic Ca^2+^ increase by activating L-type voltage-gated Ca(^2+^) channels and phospholipase C (PLC) to generate diacylglycerol (DG) and inositol trisphosphate (IP3), which further regulates the contraction and growth of VSMCs 42. In this investigation, we verified that VSMCs cytoplasmic calcium levels were clearly elevated after exposure to Ang II, and a calcium chelator significantly suppressed Ang II-induced remodeling in VSMCs, suggesting that increased cytoplasmic calcium induced by Ang II exposure was a critical factor leading to vascular remodeling in hypertension. These findings were consistent with those reported by Touyz RM et al., which stated that Ca^2+^ in VSMCs determines the contractile state and activity of various Ca^2+^-dependent proteins and transcription factors^43^. These factors and proteins control cellular function and phenotype. The Ca^2+^ release from ER is stimulated by Ang II through two ER membrane receptors, specifically the Ca^2+^-permeable ion channels RYR2 and IP3R^34^. Ang II insult significantly increased the protein levels of both RYR2 and IP3R in VSMCs. Calcium ions from the cytoplasm are pumped into the sarcoplasmic reticulum lumen by a membrane transporter and calcium pump SERCA, which promotes VSMC relaxation. According to previous research, the phosphoprotein phospholamban (PLN), found in the sarcoplasmic reticulum, acts as a reversible regulator of SERCA2a activity. Dephosphorylated PLN suppresses SERCA2a activity, while phosphorylated PLN reverses this inhibition^44^. When calcium levels in the cytoplasm rise, it binds to calmodulin and activates CaMKII, leading to CaMKII phosphorylation. We observed that Ang II exposure significantly activated CaMK II but reduced the phosphorylation levels of PLN and inactivated SERCA. Irisin overexpression/supplementation significantly reduced the cytoplasmic calcium levels in Ang II-challenged VSMCs by decreasing RYR2 and IP3R-dependent ER Ca^2+^ release, as well as increasing PLN phosphorylation levels and activating SERCA, followed by a significant decrease in CaMK II activity.

Cellular Ca^2+^ signaling is dynamic, multifactorial, and complex, and it plays an essential role in many physiological processes 22, 23. Maintaining Ca^2+^ homeostasis is crucial for cellular survival and function^22^, with the ER serving as the primary intracellular Ca^2+^ reservoir^24^. The imbalance of ER homeostasis can lead to the depletion of Ca^2+^ in ER and ER stress, which are associated with the development and progression of hypertensive vascular remodeling 26, 27. We observed that Ang II-induced ER stress in aortic tissues was worsened by *FNDC5* deficiency but alleviated by *FNDC5* overexpression. Furthermore, Ang II-induced remodeling in VSMCs, including proliferation, migration, and phenotypic transition, was significantly suppressed by an ER stress inhibitor 4-PBA, indicating a crucial player of Ang II-induced ER stress in vascular remodeling. Interestingly, irisin supplementation or a selective calcium chelator (BAPTA-AM) significantly reduced ER stress and adaptive remodeling in Ang II-treated VSMCs, suggesting that irisin suppresses hypertensive vascular remodeling by reducing ER stress and modulating cytoplasmic Ca^2+^ levels. The findings indicate that irisin is beneficial in maintaining ER homeostasis in VSMCs. It achieves this by inhibiting ER Ca^2+^ release through down-regulation of the expression levels of the Ca^2+^-permeable ion channels RYR2 and IP3R. Additionally, irisin promotes the re-uptake of cytoplasmic Ca^2+^ into the sarcoplasmic reticulum by activating the calcium pump SERCA. This, in turn, suppresses Ca^2+^-dependent ER stress. Additionally, complex pathophysiological changes occur in hypertensive vascular remodeling, including increased medial wall thickness and medial wall/lumen ratios. These changes lead to the encroachment of the medial wall into the lumen, resulting in increased vascular resistance. Previous studies have shown that irisin inhibited oxidative stress and activation of NLRP3 inflammasomes in Ang II-treated VSMCs by activating AMPK-SIRT1 signaling pathway^38^ as well as decreased blood pressure levels by alleviating endothelial dysfunction in mesenteric arteries of hypertensive rats via activating the AMPK-Akt-eNOS-NO signaling pathway^20^. These findings indicate that irisin's effects on hypertension and vascular remodeling may be mediated through its actions on endothelial cells and VSMCs, but further research is needed to fully understand these mechanisms.

Irisin, a hormone-like polypeptide, is a recently discovered to bind to its known receptor, integrin αV/β5^10^, and mediates intracellular signaling cascades through AMPK, PI3K/Akt, and members of the MAPK family (such as ERK, p38, and JNK)^9^. This hormone plays a crucial role in regulating glucose homeostasis, promoting browning of white fat, and improving cardiovascular injury. However, it is unclear whether irisin directly regulates the development of hypertensive vascular remodeling by binding to its receptors and transducing post-receptor intracellular signals. We found that exogenous irisin alone-treatment did not obviously affect the activities of AMPK, AKT, ERK, p38, or JNK. Supplementation of irisin activated AMPK and suppressed p38 in Ang II-challenged VSMCs. However, it did not significantly affect the activities of other kinases. This indicates that irisin provides protection against hypertensive vascular remodeling by acting through the AMPK and p38 signaling pathways. Furthermore, the suppression of Ang II-induced pathological remodeling changes in VSMCs (such as proliferation, migration, and phenotypic transformation) by irisin was significantly reduced by various factors. These factors include an αV/β5 integrin antibody, a specific AMPK inhibitor called compound C, and a p38 activator known as HY-W007355. These findings suggest that irisin counteracts the impairment of Ang II in VSMCs by binding to αV/β5 integrins. This binding subsequently activates the AMPK pathway while inhibiting the p38 pathway. Interestingly, ER stress was also involved in the cardiomyocyte damage in Ang II-induced hypertensive mice 45. Cardiomyocytes are known to be the cells with the richest mRNA expression of FNDC5 and are also a major source of circulating irisin 8. The crosstalk of Ang II-induced ER stress between cardiomyocytes and VSMCs involves the communication and interaction between these two cell types in response to ER stress. Ang II treatment induced an increase in ER stress in cardiomyocytes 45, which further affected the secretion of cytokines by cardiomyocytes. Irisin, as one of the essential cytokines derived from cardiomyocytes 8, may be involved in mediating the regulation of VSMC function by stressed cardiomyocytes. The complex and sophisticated regulation that exists between the hearts and the blood vessels deserves further study.

## 5. Conclusions

**Figure 10:**
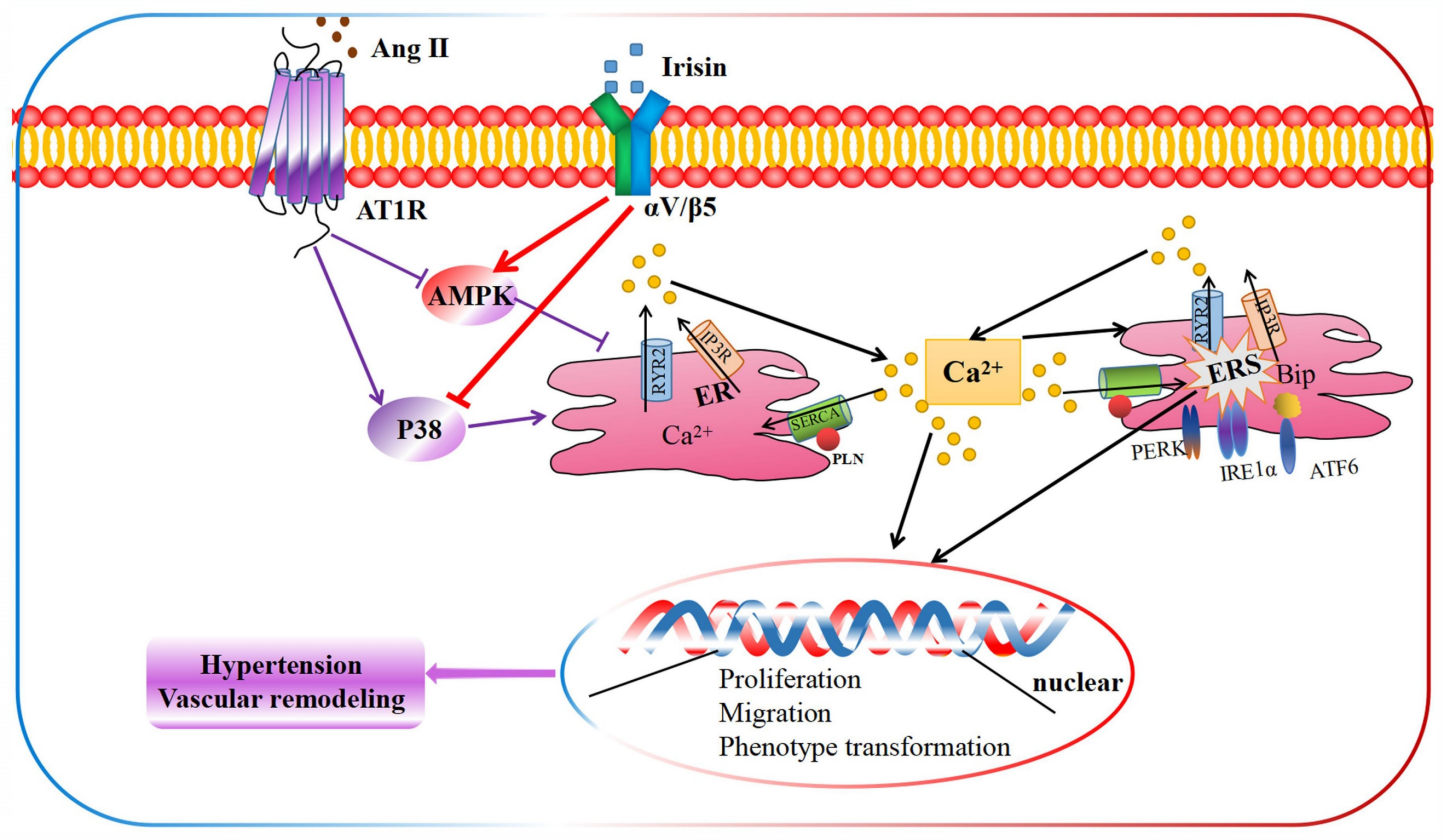
The schematic diagram illustrates the molecular mechanisms underlying irisin-mediated protection of vascular remodeling in Ang II-induced hypertension. Our study has revealed a unique mechanism through which irisin inhibits hypertension and vascular remodeling by stabilizing intracellular calcium homeostasis and down-regulating calcium-dependent ERS. Specifically, irisin binds to its receptor αV/β5 in the VSMC membrane and modulates intracellular kinases by activating AMPK and suppressing p38, thereby attenuating remodeling in Ang II-induced VSMCs.

Our study demonstrates an unprecedented mechanism through which irisin mitigates hypertension and secondary vascular remodeling by improving ER homeostasis in VSMCs. This involves inhibiting the Ca^2+^ release from ER and promoting the re-absorption of Ca^2+^ into ER, thereby alleviating Ca^2+^-dependent ER stress (Fig. 10). These findings provide conclusive evidence that irisin, its receptors, and their post-receptor signaling pathways (such as AMPK and p38) are crucial in the development of hypertension and vascular remodeling. Additionally, these results uncover the previously unknown roles of Ca^2+^ homeostasis and ER stress in irisin-induced AMPK activation and p38 inhibition during the progression of hypertensive vascular remodeling. These novel discoveries have the potential to help the development of innovative treatments for managing vascular remodeling in hypertensive diseases.

## Supplementary Material

Supplementary figures.

## Figures and Tables

**Table 1:** Primer sequences for real-time PCR amplification.

Gene name	Forward (5'-3')	Reverse (5'-3')
GAPDH	CTGCACCACCAACTGCTTAG	GGGCCATCCACAGTCTTCT
irisin	AGGATGAAGTGGTCATTGGCTTTG	CCTTGTTGTTATTGGGCTCGTTG
SMA	AGAGCAAGAGAGGGATCCTGA	GTCGTCCCAGTTGGTGATGAT
SM22	TGAAGAAAGCCCAGGAGCAT	TGCTTCCCCTCCTGCAGTT
calponin	ACCAAGCGGCAGATCTTTGA	CATCTGCAAGCTGACGTTGA
caldesmon1	GGAGGCTGATCGAAAAGCTA	AGCTTCTGCCCTTCTCCTTT

## Abbreviations

Ang II: angiotension II
CaMK II: calmodulin-dependent protein kinase II
DBP: diastolic blood pressure
ECM: extracellular matrix
ER: endoplasmic reticulum
ERS: endoplasmic reticulum stress
*FNDC5*: fibronectin type III domain containing protein 5
IP3R: inositol 1,4,5-trisphosphate receptor
MABP: mean arterial blood pressure
SBP: systolic blood pressure
SERCA: sarcoplasmic reticulum calcium-ATPase
SM22: smooth muscle protein of 22 kDa
SMA: SM α-actin
SOCE: store-operated calcium entry
OV: overexpression
PLC: phospholipase C
PLN: phospholamban
RyR2: type 2 ryanodine receptor
UPR: unfolded protein response
VSMC: Vascular smooth muscle cells
