# Supplementary material for: Irisin attenuates vascular remodeling in hypertensive mice induced by Ang II by suppressing Ca2+-dependent endoplasmic reticulum stress in VSMCs
Source: Int J Biol Sci. 2024 Jan 1;20(2):680–700. doi: 10.7150/ijbs.84153 (PMC10758105; doi:10.7150/ijbs.84153)

Supplementary Figure legends

Supplementary Figure 1. PCR assays was used to identify the genotype of *FNDC5* knockout (KO) and overexpression (OV) mice.
A. The genotyping of irisin-KO mice.
B. The genotyping of irisin-OV mice.

Supplementary Figure 2. Irisin deficiency or overexpression had no significant effect on lumen radius of aortas in mice infused with saline or Ang II (490 ng/min/kg) for 4 weeks.
A. The H-E staining image of thoracic aorta tissues from irisin-KO, irisin-OV and WT mice challenged with saline or Ang II (490 ng/min/kg) for 4 weeks. B. Lumen radius of aortas from irisin-KO, irisin-OV and WT mice challenged with saline or Ang II (490 ng/min/kg) for 4 weeks (n = 8/group).
The data were presented as mean ± S.E.M., and statistical analyses were performed by one way ANOVA. *P < 0.05.

Supplementary Figure 3. The changes in the expression levels of αV/β5 and intracellular kinases in VSMCs treated with irisin (20 nM) or Ang II (1 μM) alone, or Ang II (1 μM) in combination with irisin (20 nM), respectively.
A. Changes in mRNA levels of αV and β5 in VSMCs treated with irisin (20 nM) or Ang II (1 μM) alone, or Ang II (1 μM) in combination with irisin (20 nM) for 24 h, respectively (n=3/group).
B. Changes in the expression levels of phosphorylation and total kinase proteins, including AKT, ERK and JNK, in VSMCs challenged with irisin (20 nM) or Ang II (1 μM) alone, or Ang II (1 μM) in combination with Ang II (1 μM) for 24 h, respectively (n=3/group).
The data were presented as mean ± S.E.M., and statistical analyses were performed by one way ANOVA. **P < 0.01.

Supplementary Figure 4. The mRNA and protein expression distribution of *FNDC5* in smooth muscle.
A. The mRNA expression overview of *FNDC5* in different organs from website of "The Human protein Alats".
B. The protein expression overview of *FNDC5* in different organs from website of "The Human protein Alats".

Supplement Figure 1

A

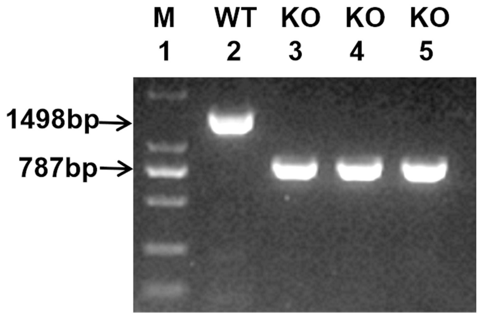

B

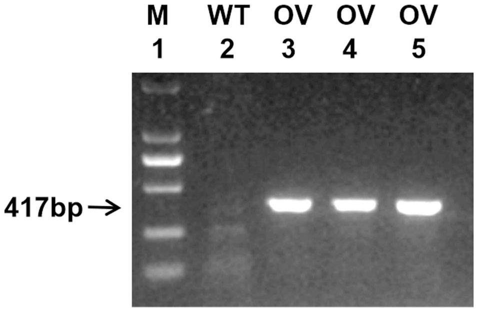

Supplement Figure 2

A

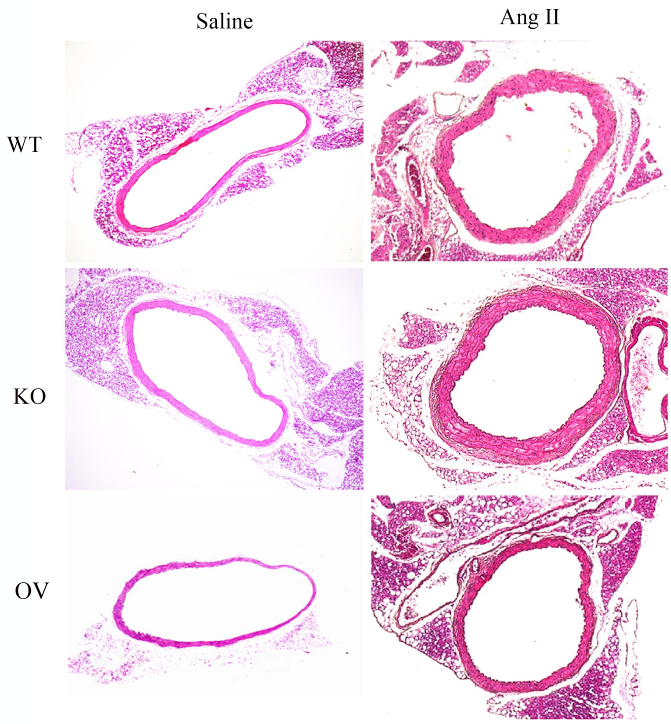

B

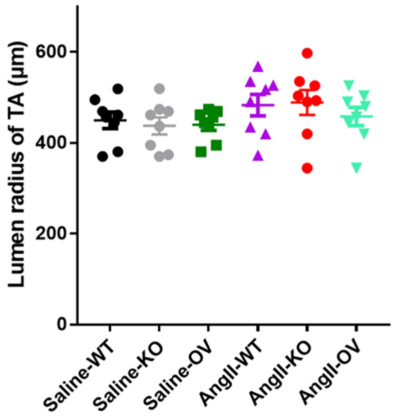

Supplement Figure 3

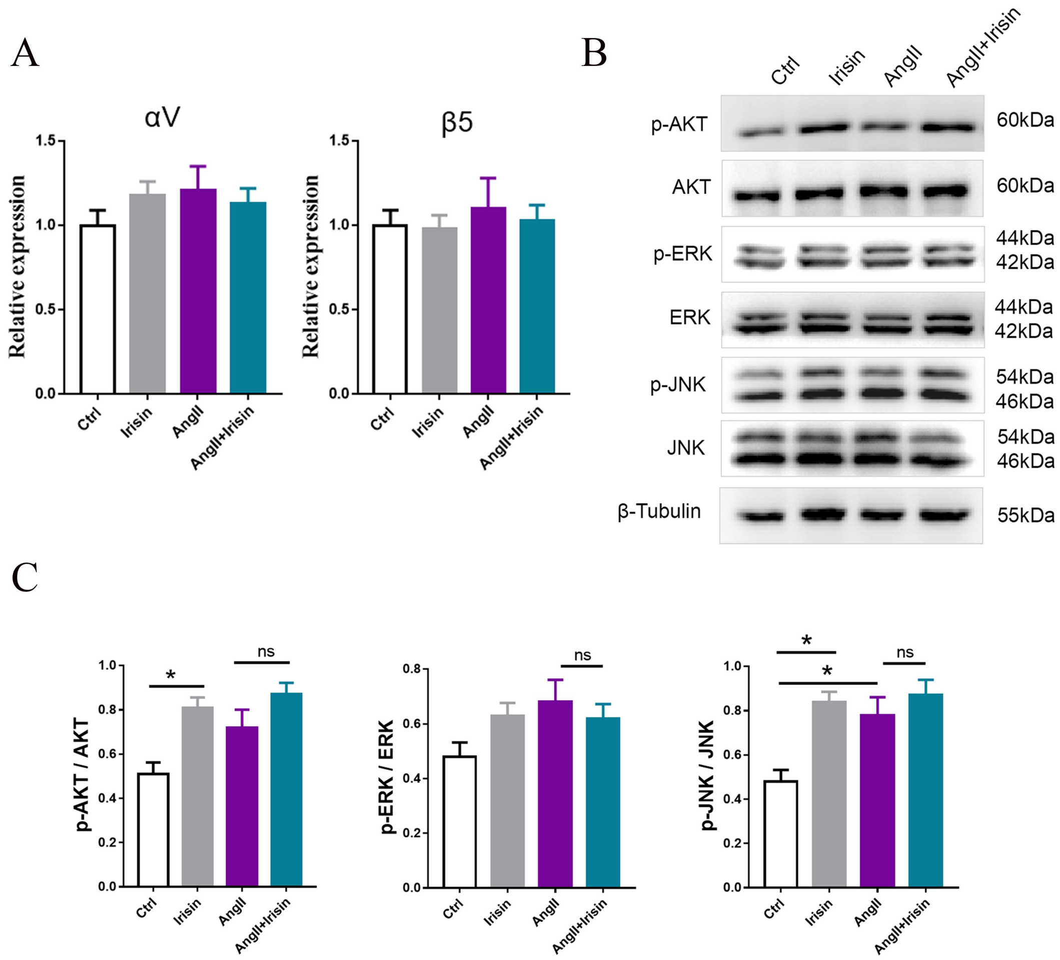

Supplement Figure 4

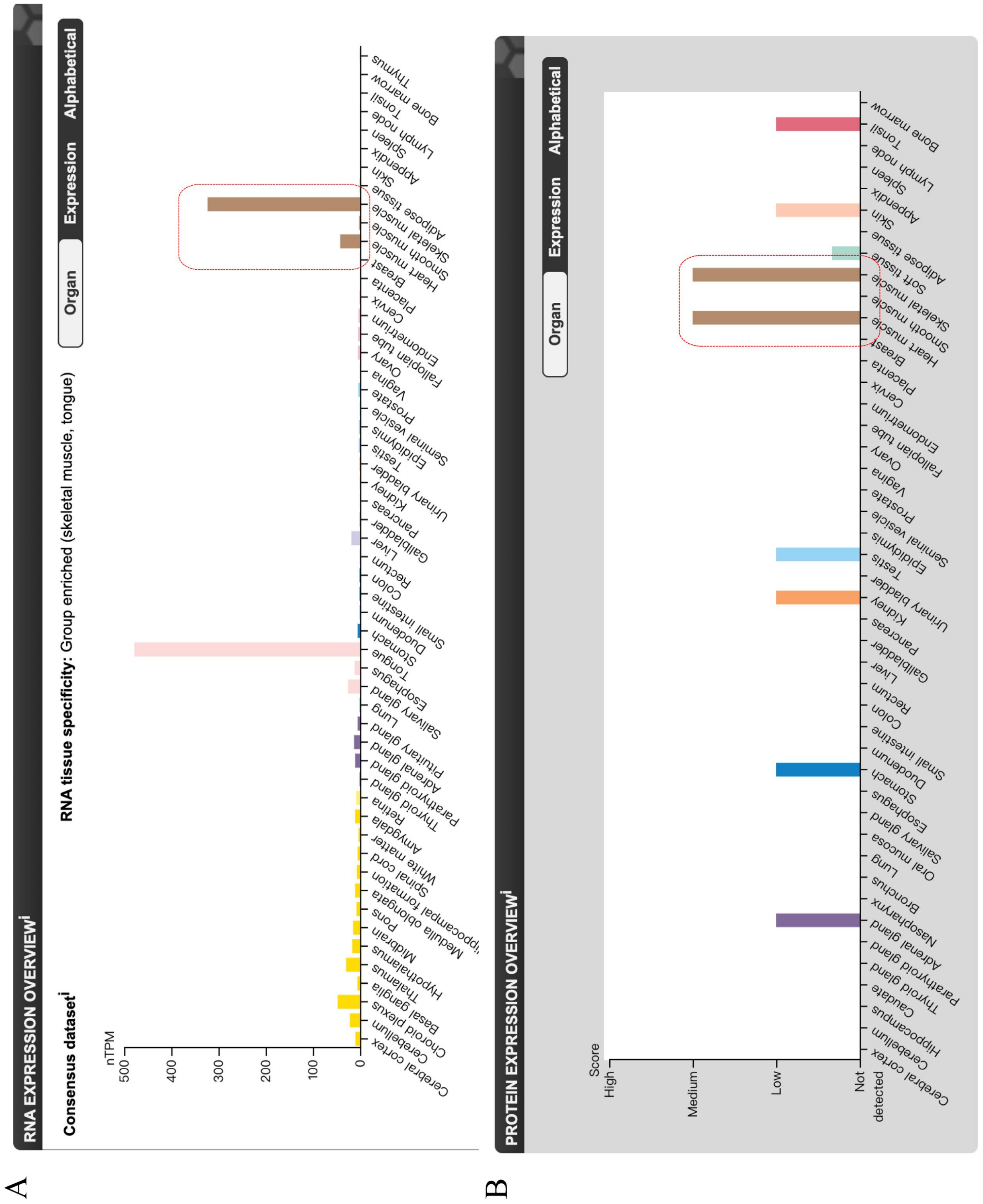

Supplement: Supplementary file 1 — Supplementary figures. [file ijbsv20p0680s1.pdf]
